# Retinal Pathology and Synucleinopathy in the Visual Pathway of α‐Synuclein Preformed Fibril Mouse Model of Parkinson's Disease

**DOI:** 10.1002/brb3.71489

**Published:** 2026-05-14

**Authors:** Qin Zhang, Xin‐Yi Qian, Tao Peng, Xiao‐Ou Hou, Zhen‐Yuan Ma, Jiang Huang, Li‐Fang Hu, Wei‐Feng Luo

**Affiliations:** ^1^ Department of Neurology Second Affiliated Hospital of Soochow University Suzhou Jiangsu China; ^2^ Department of Neurology, The Affiliated Changsha Central Hospital, Hengyang Medical School University of South China Changsha Hunan China; ^3^ Department of Ophthalmology Second Affiliated Hospital of Soochow University Suzhou Jiangsu China; ^4^ School of Future Science and Engineering Soochow University Suzhou Jiangsu China; ^5^ Department of Neurology and Clinical Research Center of Neurological Disease The Second Affiliated Hospital of Soochow University Suzhou Jiangsu China; ^6^ Jiangsu Key Laboratory of Drug Discovery and Translational Research for Brain Diseases, Institute of Neuroscience Soochow University Suzhou Jiangsu China; ^7^ Suzhou Key Laboratory of Geriatric Neurological Disorders Taicang Affiliated Hospital of Soochow University Suzhou Jiangsu China

**Keywords:** α‐synuclein, Parkinson's disease, retina, visual dysfunction, visual pathway

## Abstract

**Background:**

Visual dysfunction is a common nonmotor manifestation of Parkinson's disease (PD) that may precede motor symptoms. This study aimed to characterize the early preformed fibril (PFF) mouse model of PD.

**Methods:**

Male C57BL/6J mice received intrastriatal injections of α‐synuclein (α‐syn) PFFs or phosphate‐buffered saline. Visual function was evaluated at 3 and 6 months postinjection using pattern visual evoked potentials (PVEPs) and the visual cliff test. Retinal morphology and protein expression were assessed by hematoxylin–eosin staining, immunofluorescence, and Western blot analysis for phosphorylated α‐syn (pS129), tyrosine hydroxylase (TH), glial fibrillary acidic protein (GFAP), and Iba1. Pathological α‐syn distribution in the visual pathway and association cortices was examined by fluorescence microscopy.

**Results:**

At 3 months, PFF‐injected mice showed prolonged PVEP latency and reduced amplitude, indicating early visual pathway dysfunction, which worsened by 6 months. Retinal structure was preserved, but *p*‐α‐syn accumulation appeared in ganglion cells, accompanied by reduced TH expression and activation of microglia and Müller glia. The pSer129‐immunoreactive structures were detected in the visual cortex and visual association cortices, including frontal, parietal, temporal, and amygdaloid regions.

**Conclusions:**

Functional and pathological alterations in the visual system emerge before motor deficits in α‐syn PFF‐injected mice. Early retinal and cortical synucleinopathy may underlie prodromal visual dysfunction and serve as potential biomarkers for early PD diagnosis.

## Graphical abstract

Schematic summary of visual electrophysiology and retinal and brain pathology. The diagram integrates PVEP abnormalities, retinal pathology, and brain pathology in PFF‐injected mice. Based on behavioral outcomes, the three groups were categorized as control, prodromal PD, and PD, corresponding to the PBS, 3‐month PFF (3 MPI), and 6‐month PFF (6 MPI) groups, respectively. In prodromal PD mice, reductions in PVEP P100 latency and amplitude, decreased retinal TH expression, and microglial activation were already detectable. *p*‐α‐Syn immunoreactivity was observed along the visual pathway (retina and visual cortex), as well as in higher‐order visual association areas (prefrontal, parietal, and temporal association cortices) and the amygdala. These electrophysiological and pathological changes were more pronounced in PD mice. BLA, basolateral nucleus of amygdale.

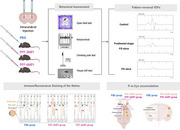

**Abbreviations**
α‐syn: α‐synuclein
BLA: basolateral nucleus of amygdale
DA: dopamine
DACs: dopaminergic amacrine cells
DNs: dopaminergic neurons
ERG: electroretinography
FC: frontal motor cortex
GCL: ganglion cell layer
GFAP: glial fibrillary acidic protein
Hip: hippocampus
INL: inner nuclear layer
IPL: inner plexiform layer
LGN: lateral geniculate nucleus
OC: optic chiasma
OCT: optical coherence tomography
ON: optic nerve
ONL: outer nuclear layer
OPL: outer plexiform layer
OT: optic tract
PC: parietal cortex
PD: Parkinson's disease
PFF: preformed fibrils
RGCs: retinal ganglion cells
RNFL: retinal nerve fiber layer
SN: substantia nigra
STR: striatum
TeA: temporal association cortex
TH: tyrosine hydroxylase
VC: visual cortex
VEPs: visual evoked potentials

## 1 Introduction

Parkinson's disease (PD) represents a prevalent age‐associated neurodegenerative disorder of the central nervous system, often developing gradually and progressing irreversibly, ultimately leading to marked deterioration in patients’ daily functioning and life quality (Harrison and Luciano 2021). Its core pathological hallmarks comprise the selective degeneration of dopaminergic neurons (DNs) within the nigrostriatal circuitry and the misfolding and aggregation of α‐synuclein (α‐syn) (Sharma et al. 2025). The aberrant accumulation of α‐syn has been identified as a pivotal contributor to PD pathophysiology, implicated in driving dopaminergic neurotoxicity, compromising the integrity of the blood–brain barrier, and amplifying neuroimmune responses (Shulman et al. 2011).

Visual impairments are frequently observed among PD patients and represent a predominant category of nonmotor symptoms. Epidemiological investigations suggest that nearly 78% of individuals with PD experience varying degrees of visual dysfunction, including compromised acuity, diminished contrast sensitivity, alterations in color perception, impaired motion detection, and visual hallucinations—many of which may present prior to classical motor signs (Weil et al. 2016; Ortuño‐Lizarán et al. 2020; Vidailhet 2024). Recent studies using visual evoked potentials (VEPs) and electroretinography (ERG) have revealed attenuated retinal electrical activity in early‐stage PD patients (Casciano et al. 2024; Sipos‐Lascu et al. 2024). Structural imaging via optical coherence tomography (OCT) has consistently revealed reduced thickness in the retinal nerve fiber layer (RNFL), a metric often associated with clinical disease severity (Poveda et al. 2024). Functional neuroimaging has shown reduced metabolic activity in posterior cortical regions, such as the occipital, parietal, and parts of the temporal lobes, in PD patients (Armstrong 2017). Moreover, abnormal connectivity of visual‐related resting‐state networks has been found (Baik et al. 2024). Postmortem analyses provide further evidence of α‐syn pathology within the retina and optic nerve (ON), often paralleling the burden of synucleinopathy in corresponding brain structures (Li et al. 2023; Hart de Ruyter et al. 2023). These studies demonstrate the potential of changes in the functions and structures of the visual pathways, including the retina, in the early diagnosis and disease assessment of PD. However, the underlying mechanisms remain unclear, and the specificity of these visual changes as PD biomarkers requires further validation. Additionally, the propagation of pathological α‐syn within the visual system has yet to be fully elucidated.

Although current studies have attempted to characterize the morphological and functional alterations in the visual pathways of patients with PD, the ability to investigate visual system changes during the prodromal phase remains limited due to the inherent constraints of prospective clinical studies. In contrast, animal models of PD offer a powerful platform to overcome these challenges and enable the detailed examination of early pathophysiological changes. To date, only a limited number of studies have described retinal pathology in PD animal models. For instance, the MPTP neurotoxin model (Tran et al. 2022) and adeno‐associated virus (AAV)‐mediated overexpression of human wild‐type α‐syn (Marrocco et al. 2020) have demonstrated reduced retinal electrophysiological responses, visual impairment, and loss of retinal dopaminergic amacrine cells (DACs). Similarly, in α‐syn transgenic mice (TgM83), accelerated α‐syn accumulation (Mammadova et al. 2021) and severe degeneration of photoreceptor cells (Xu et al. 2024) have been documented. However, most of these studies have focused primarily on retinal alterations and have paid little attention to pathological changes along the entire visual pathway, particularly during the prodromal phase.

α‐Syn preformed fibrils (PFFs) serve as seeds that can induce the aggregation of endogenous α‐syn within cells, exhibiting prion‐like propagation properties. When injected via stereotactic delivery into the brain, α‐syn PFFs can recapitulate the progressive α‐synucleinopathy and motor symptoms observed in PD, thereby providing a more accurate model of disease progression compared to toxin‐ or transgene‐based models (Paumier et al. 2015; Patterson et al. 2019). This makes the α‐syn PFF model a valuable tool for investigating the pathogenesis and early diagnosis of prodromal PD. In this study, we employed striatal stereotaxic injection of α‐syn PFFs to establish a PD mouse model. We assessed visual function, retinal morphology, electrophysiological activity along the visual pathway, and the pattern of pathological α‐syn accumulation during both the prodromal and symptomatic stages. Our results revealed that abnormalities in visual electrophysiology, downregulation of tyrosine hydroxylase (TH) expression, and glial activation occurred prior to the onset of motor symptoms. Furthermore, pSer129‐immunoreactive structures were detected in the retina and visual‐related brain regions. These findings suggest that functional and pathological alterations in the visual system may serve as potential early biomarkers for PD. Notably, we also observed pathological α‐syn accumulation in higher‐order visual processing areas—including the visual cortex (VC), frontal, parietal, and temporal association cortices, as well as the amygdala—during the premotor phase. This may underlie the emergence of complex visual disturbances and highlights the involvement of widespread visual circuitry in early PD.

## 2 Materials and Methods

### 2.1 Production of Recombinant α‐Syn and Preparation of Human α‐Syn PFFs

α‐Syn PFFs were generated as previously described (Volpicelli‐Daley et al. 2014). In brief, wild‑type human α‐syn protein was heterologously produced using *Escherichia coli* BL21 (DE3) cells transformed with a pET‐28b (+) vector encoding the full‐length human α‐syn sequence. Following transformation, cultures were expanded in Luria–Bertani (LB) broth and protein expression was triggered by the addition of 1 mM isopropyl β‐*D*‐1‐thiogalactopyranoside (IPTG). Bacterial cells were collected by centrifugation at 5000 rpm for 5 min and resuspended in Tris‐based lysis buffer (150 mM Tris, pH 8.0, supplemented with 10 mM EDTA and 150 mM NaCl). Cell disruption was achieved via ultrasonication, and lysates were clarified by centrifugation at 13,000 rpm for 15 min. The supernatant was dialyzed with 10 mM Tris (pH 7.6), 50 mM NaCl, and 1 mM EDTA. The supernatant containing soluble α‐syn was subjected to nickel column purification using a His‐tag purification kit (Beyotime, P2226, China). Specifically, the supernatant was mixed with 50% BeyoGold His‐tag resin and incubated on a shaker at 4°C for 1 h. The column was subsequently washed six times with wash buffer, followed by six elutions with elution buffer, and then rinsed twice with ddH_2_O. The resin was finally stored in a 20% ethanol solution (prepared in phosphate‐buffered saline [PBS]) at 4°C. The purified protein was transferred to a 3 kDa ultrafiltration tube and concentrated by centrifugation at 7500 rpm for 30 min at 4°C. The concentrated protein retained in the upper filter was subjected to two additional rounds of concentration under the same conditions, after which 2 M NaCl was added, and the sample was stored at room temperature. Endotoxins were removed using an endotoxin removal kit (ToxinEraserTM, GenScript, L00338, China). Following bicinchoninic acid (BCA) protein quantification, the monomeric protein was diluted with PBS to a final concentration of 5 mg/mL.

For fibrillization, purified monomeric α‐syn was subjected to constant agitation (1000 rpm) at 37°C for 7 days in an Eppendorf Thermomixer, facilitating the in vitro formation of fibrillar assemblies. Prior to experimental application, the resulting fibrils were diluted and fragmented into short PFFs via probe sonication at 10% amplitude for 30 s with alternating pulses (0.5 s on/off). The structural integrity and fibrillar morphology of α‐syn PFFs were confirmed using transmission electron microscopy (TEM).The structural integrity and morphology of the PFFs used were verified by TEM, as previously described (Volpicelli‐Daley et al. 2014; Pei et al. 2024)

### 2.2 Animals and Stereotaxic Injection Into the Striatum (STR)

All experimental procedures were approved by the Institutional Animal Care and Use Committee (IACUC) and were conducted in accordance with institutional guidelines. Male wild‐type C57BL/6J mice (8 weeks old) were housed in a specific pathogen‐free (SPF) animal facility at 22°C, with ad libitum access to food and water under a 12‐h light/dark cycle. A maximum of five animals were housed per cage. Mice were randomly assigned to three experimental groups: PBS‐injected control group, 3‐month postinjection group (3 MPI), and 6‐month postinjection group (6 MPI). Initially, 12 mice were injected in each of the three groups. All animals underwent behavioral testing and pattern visual evoked potential (PVEP) recording before being used for histological and protein analyses. In the supplementary experiment, six mice were added to each group for the analysis of brain sagittal *p*‐α‐syn immunofluorescence staining.

Mice were subcutaneously injected with extended‐release buprenorphine suspension (XR, 3.25 µg/g) 30 min before the operation, and anesthetized intraperitoneally with Avertin (250 µg/g). Following the successful induction of anesthesia, mice were placed in a stereotaxic frame. After disinfection with iodine and alcohol, the scalp was incised and retracted to expose the skull. A cotton swab soaked in 30% hydrogen peroxide was used to clean the skull surface and identify bregma. Using bregma as the reference point, bilateral striatal coordinates were determined as follows—anteroposterior (AP): +0.2 mm; mediolateral (ML): ±2.0 mm; dorsoventral (DV): +2.6 mm. A small burr hole was drilled at each site, and a microsyringe was slowly lowered into the target region. A total volume of 2 µL α‐syn PFF solution (2.5 µg/µL) or PBS was injected per hemisphere at a rate of 0.2 µL/min. After injection, the needle was left in place for 5 min before being slowly withdrawn. The surgical site was disinfected with povidone‐iodine, sutured, and treated with erythromycin ointment to prevent infection. Mice were identified with ear tags, placed on a heating pad for recovery, and returned to the animal holding room once fully awake for subsequent experimentation.

### 2.3 Behavioral Assessments

#### 2.3.1 Open Field Test

To evaluate spontaneous locomotor activity and general exploratory behavior, mice were assessed using the open field paradigm within a cubic enclosure measuring 40 × 40 × 40 cm. Prior to testing, animals were habituated to the experimental environment for a minimum of 30 min to minimize stress‐related variability. The apparatus was sanitized with 70% ethanol between sessions to eliminate residual olfactory traces and subsequently air‐dried. Each mouse was gently introduced into a corner along the outer boundary of the arena, and behavioral activity was continuously monitored for a 10‐min period using an overhead video camera.

#### 2.3.2 Rotarod Test

To assess motor coordination and balance, mice were subjected to rotarod testing. The training was performed once daily for 3 consecutive days prior to testing, with three trials per day at a constant speed of 20 rpm for approximately 3 min per trial. During the test phase, the rotarod was programmed to accelerate from 4 to 40 rpm over a 5‐min period. The latency to fall and the rotation speed at the time of fall were automatically recorded.

#### 2.3.3 Climbing Pole Test

Motor coordination and balance were assessed using a vertical pole apparatus, consisting of a 50 cm‐high rod (1 cm in diameter) topped with a 2.5 cm‐diameter sphere to facilitate placement. Mice underwent 3 days of habituation to minimize novelty‐induced variability. For each trial, the mouse was positioned facing upward on the top of the pole and gently prompted to reorient downward. The duration required to complete the turn and descend to the platform base was recorded. Each subject completed three trials, and the average descent time was used for further evaluation.

#### 2.3.4 Grip Strength Test

Forelimb muscle strength was quantified using a calibrated grip strength meter. Mice were allowed to firmly grasp the instrument's mesh grid with their forepaws while being steadily pulled backward by the tail until they released their grip. The peak force generated during each pull was recorded automatically. Measurements were repeated three times per animal, and the mean force was used for statistical comparisons.

#### 2.3.5 Visual Cliff Test

The visual cliff apparatus consisted of a transparent acrylic box (80 × 80 × 25 cm) positioned so that half extended beyond the edge of the laboratory bench. A checkerboard pattern was placed beneath the floor, creating an illusion of depth on one side. The apparatus was cleaned and deodorized with ethanol before each trial. Mice were placed along the midline of the floor, and the time spent on the “deep” side of the platform was recorded as a measure of visual perception.

### 2.4 PVEP Measurement

The PVEP was completed using the visual electrophysiological examination system (GT‐2000NV). Before the test, the mice were placed in a dark room for 1 h. They were then anesthetized intraperitoneally with 250 µg/g of Avertin and fixed in a stereotaxic frame with a heating pad maintaining rectal temperature at 37.0°C ± 0.5°C. The corneas were protected with ophthalmic gel (Viscotears). After scalp disinfection and exposure of the skull, small cranial windows (1.5 mm diameter) were drilled bilaterally over the VC (4 mm posterior to bregma, 3 mm lateral from the midline), leaving the dura intact. Metal electrodes were placed on the dura over the VC and secured with tape, without damaging the brain tissue. The reference electrode was inserted subcutaneously, placed between the skin and the nasal bone. The ground electrode was inserted subcutaneously into the tail of the mouse. The resistance between the recording electrode, the reference electrode, and the ground electrode was measured, and the impedance was less than 10 kΩ. The system display was used to provide a reversing checkerboard stimulus. The screen was placed about 15 cm in front of the eyes. The non‐test eye was blocked. A black and white reversing checkerboard stimulus was given, with a spatial frequency of 0.07 cycles/degree, a temporal frequency of 1 Hz, a brightness of 100%, a contrast of 97%, a passband of 1–300 Hz, and 80 superimpositions. The PVEP response signal was observed for 300 ms after averaging. The amplitude and latency of the P100 wave of this PVEP signal were measured. Each mouse was measured three times and the average was taken. There was a 10‐min interval between each two measurements. All mice from the three experimental groups were euthanized by transcardial perfusion immediately after PVEP data collection. Tissues were harvested for subsequent analyses, and the animals were not allowed to recover from anesthesia.

### 2.5 Tissue Collection

At designated time points postinjection, mice were anesthetized with an intraperitoneal injection of Avertin (250 µg/g) and perfused transcardially with PBS. Retinas, ON, and brains were rapidly dissected on ice. Samples designated for protein analysis were snap‐frozen in liquid nitrogen and stored at −80°C. For histological analyses, eyeballs and brain tissues were fixed in 4% paraformaldehyde (PFA) at 4°C for subsequent cryosectioning and immunohistochemistry.

### 2.6 Hematoxylin and Eosin (H&E) Staining of the Retina

Following enucleation, ocular tissues were immersion‐fixed in 4% PFA at 4°C overnight. Samples were subsequently processed for paraffin embedding, and sagittal sections (8 µm thick) encompassing the ON head were obtained using a rotary microtome. After mounting onto glass slides, the sections underwent standard H&E staining. Stained samples were air‐dried and sealed with coverslips for preservation. Digital images were captured employing a laser scanning confocal microscope (LSM 900, Zeiss), and quantitative morphological analyses were implemented utilizing ImageJ software (NIH, USA). Of the seven mice used for retinal H&E staining analysis, some samples were excluded due to tissue damage, resulting in six samples per group for final analysis. Cell density (cells/mm^2^) was quantified in the ganglion cell layer (GCL), inner nuclear layer (INL), outer nuclear layer (ONL), and the full retinal thickness across three retinal regions—posterior pole, equatorial region, and peripheral region. For each staining, three sections per sample were analyzed from each of the three regions, with three fields of view examined per section.

### 2.7 Immunofluorescence Staining of the Retina and ON

Perfused eyeballs and ONs were postfixed overnight in 4% PFA at 4°C, followed by dehydration in graded sucrose solutions (10%, 20%, and 30%) for 24 h each. Dehydrated tissues were embedded in optimal cutting temperature compound, transferred into embedding molds, and rapidly frozen at −80°C for cryopreservation. Sagittal cryosections of the retina and ON (12 µm thickness) were prepared utilizing a cryostat and mounted onto glass slides. Slides were air‐dried in a 37.5°C incubator for 2 h, then stored at −80°C until use. Prior to staining, sections were brought to room temperature, rinsed thrice in 1× PBS, and permeabilized/blocked in PBST (PBS + 0.3% Triton X‐100) containing 5% fetal bovine serum (FBS) for 1 h at room temperature. Following PBS washing, the sections were incubated overnight at 4°C with the following primary antibodies (diluted in PBST): anti‐phospho‐S129 α‐syn (pS129 α‐syn; 1:500, Abcam, ab51253), anti‐α‐syn (1:500, Cell Signaling Technology, 4179), anti‐NeuN (1:500, Millipore, MAB377), anti‐TH (1:800, Oasis Biopharma, OB‐PRB100), anti‐Iba1 (1:800, Oasis Biopharma, OB‐PGP049‐01), and anti‐GFAP (1:500, Santa Cruz Biotechnology, sc‐65343). After primary antibody incubation, sections were washed thrice in PBS (10 min each), followed by a 1‐h incubation at room temperature in the dark with corresponding fluorescent secondary antibodies (1:500): Alexa Fluor 488 (Molecular Probes, R37118) and Alexa Fluor 555 (Molecular Probes, R37115). Slides were washed again in PBS thrice (10 min per wash), air‐dried, and coverslipped using a DAPI‐containing mounting medium. Coverslips were sealed with nail polish and allowed to air‐dry before imaging. Fluorescent images were captured using a laser scanning confocal microscope (LSM 900, Zeiss), and image processing was performed using Zen Blue software version 3.6. Of the seven mice used for retinal histological analysis, some samples were excluded due to tissue damage, resulting in six samples per group for final analysis. Sagittal sections passing through the ON were selected for retinal analysis. For each staining, three sections per sample were analyzed from each of the three regions (posterior pole, equatorial region, and peripheral region), with three fields of view examined per section.

### 2.8 Immunofluorescence Staining of Brain Tissue

Perfused brains were postfixed in 4% PFA overnight at 4°C in 50 mL conical tubes, followed by cryoprotection in 10% and 20% sucrose solutions for 24 h each and 30% sucrose for 48 h. Dehydrated brains were embedded in optimal cutting temperature compound and frozen at −80°C, and sectioned using a cryostat. Serial coronal sections were obtained from selected brain regions, including the STR, frontal motor cortex (FC), parietal cortex (PC), VC, temporal association cortex (TeA), superior colliculus, lateral geniculate nucleus (LGN), optic chiasm (OC), optic tract (OT), and amygdala. Sections through the substantia nigra (SN) were cut at a thickness of 18 µm, while sections through other brain regions were cut at 20 µm. Every sixth section spanning the entire rostro‑caudal extent of the region of interest was collected and processed. In addition, sagittal brain sections passing through the striatal injection site were prepared at a thickness of 20 µm. Sections were collected into 24‐well plates containing PBS to remove excess OCT and washed thrice with PBS (5 min each). Sections were incubated overnight at 4°C with the following primary antibodies diluted in PBS with 0.3% Triton X‐100 (PBST): anti‐phospho‐S129 α‐syn (1:500, Abcam, ab51253), anti‐TH (1:800, Oasis Biopharma, OB‐PRB100), and anti‐dopamine transporter (DAT; 1:800, Millipore, MAB369). After primary incubation, sections were washed thrice with PBS (10 min each) and incubated for 1 h at room temperature in the dark with corresponding fluorescent secondary antibodies (1:500): Alexa Fluor 488 (Molecular Probes, R37118) and Alexa Fluor 555 (Molecular Probes, A31572). Following secondary incubation, sections were washed again in PBS (3 × 10 min), air‐dried, and mounted using a DAPI‐containing mounting medium. Coverslips were sealed with nail polish and air‐dried before imaging. Whole‐slide scans were performed using a VS200 slide scanner (Olympus), and high‐resolution fluorescence imaging was implemented utilizing a confocal laser scanning microscope (LSM 900, Zeiss). Image processing and quantification were carried out using Zen Blue software version 3.6. Of the 12 mice initially injected, seven were used for histological analysis, including DAT and TH immunofluorescence staining in the brain; all six mice from the supplementary experiment were used for *p*‐α‐syn immunofluorescence staining on sagittal brain sections. For each staining, six sections per sample were analyzed, with three fields of view examined per section. TH^+^ neuron density (cells/mm^2^) was quantified in the substantia nigra pars compacta (SNc).

### 2.9 Western Blot Analysis of Retinal Lysates

Retinal tissues were lysed on ice in a buffer solution using brief pulse sonication (1–2 s) until fully homogenized. The lysates were centrifuged at 12,000 rpm for 30 min at 4°C, and the resulting supernatants were collected for downstream protein analysis. Protein concentrations were determined with a BCA assay kit. Equal protein amounts were denatured by mixing with 2× SDS sample buffer and heating at 95°C for 10 min. Proteins were resolved via SDS‐PAGE using either 10% or 13.5% polyacrylamide gels, selected based on the molecular weight of the target proteins. Electrophoresis was performed at 90 V for 30 min followed by 120 V for approximately 90 min. Following separation, proteins were transferred onto PVDF membranes and blocked with 5% nonfat dry milk in PBST (PBS with 0.1% Tween‐20) for 1 h at ambient temperature. Membranes were then incubated overnight at 4°C with the following primary antibodies: anti‐phospho‐S129 α‐syn (1:500; Abcam, ab51253), anti‐TH (1:800; Oasis Biopharma, OB‐PRB100), anti‐GAPDH (1:500,000; Proteintech, 60004‐1‐Ig), and anti‐β‐actin (1:1000; Santa Cruze, SC‐47778). After three washes with TBST, membranes were probed with HRP‐conjugated secondary antibodies for 1 h at room temperature with gentle agitation. Five mice per group were used for Western blot analysis. Protein bands were visualized using enhanced chemiluminescence (ECL) reagents and documented using a chemiluminescence imaging system. Band intensities were quantified by densitometric analysis using ImageJ software (NIH, USA), and grayscale values were normalized and subjected to statistical evaluation. Some samples were excluded due to poor transfer quality or insufficient exposure affecting densitometric quantification, yielding a final sample size of four mice per group for Western blot analysis.

### 2.10 Statistical Analysis

All behavioral and histological analyses were performed in a blinded manner. The experimenter was unaware of group allocation until all data were collected and quantified.

All data are presented as mean ± standard error of the mean (SEM) from at least three independent experiments unless otherwise specified. Statistical analysis was done utilizing GraphPad Prism 9.2.0 (GraphPad Software Inc., La Jolla, California, USA). Unpaired Student's *t*‐test was used for comparisons between two groups, while one‐way ANOVA was used for comparisons among multiple groups. A *p*‐value less than 0.05 was deemed statistically significant. Graphs were generated utilizing GraphPad Prism 9.2.0.

## 3 Results

### 3.1 Progressive Emergence of Depressive‐Like Behavior and Motor Deficits at 3 and 6 Months Post‐Striatal PFF Injection

**FIGURE 1:**
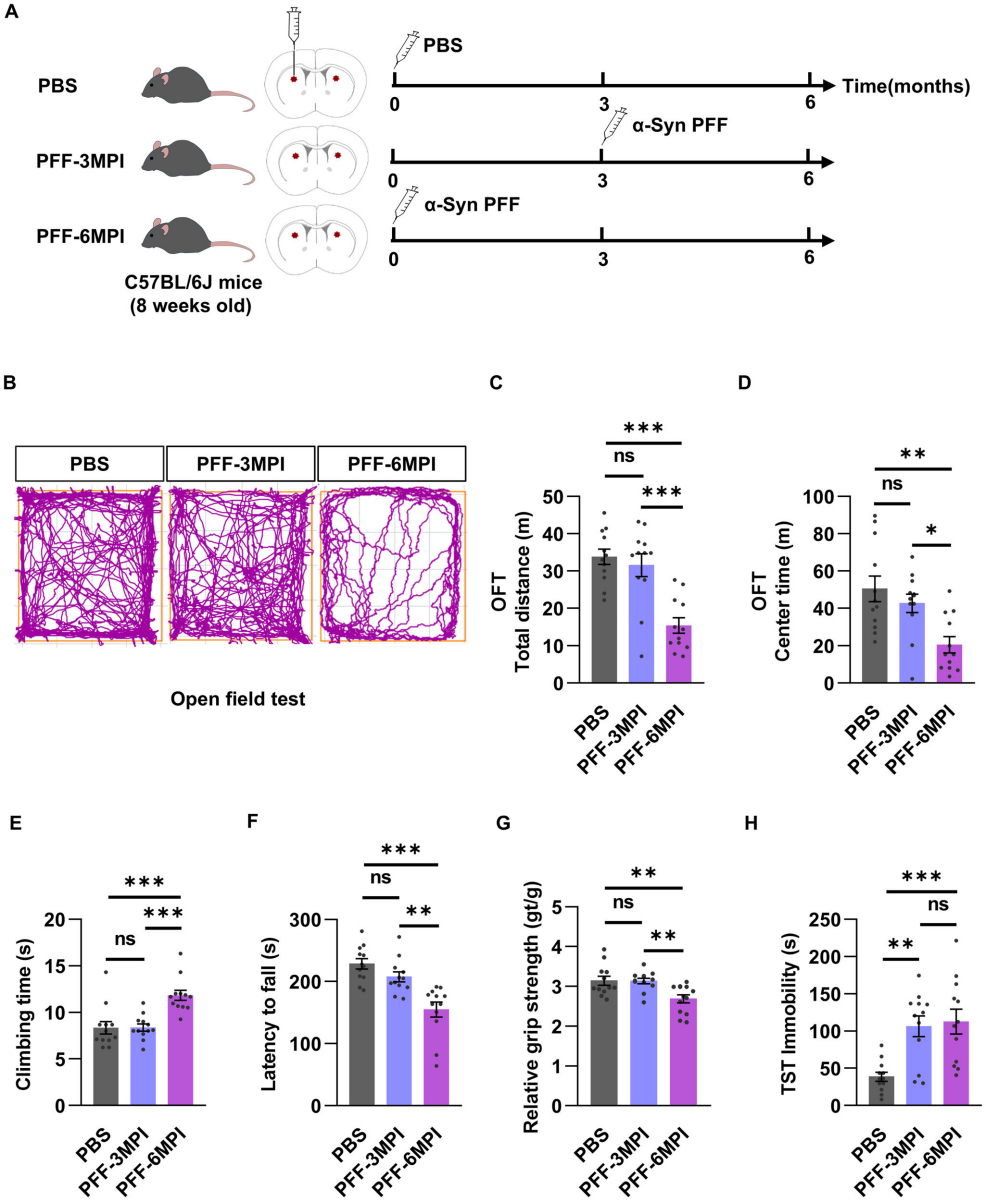
Progressive appearance of depressive‐like behavior and motor dysfunction in mice following intrastriatal injection of α‐syn PFFs. (A) Injection time schedule for the three mouse groups. (B) Representative locomotor traces in the open field test for control, 3 MPI, and 6 MPI mice. (C) Quantification of total distance traveled. (D) Time spent in the center zone of the open field arena. (E) Time required to descend in the pole test. (F) Latency to fall in the rotarod test. (G) Forelimb grip strength (normalized). (H) Immobility time in the tail suspension test. Data are presented as means ± SEM. Group sizes: PBS (*n* = 12), PFF‐3MPI (*n* = 12), PFF‐6MPI (*n* = 12). Statistical significance determined by one‐way ANOVA: *p < 0.05, **p < 0.01, ***p < 0.001.

To evaluate motor performance, we conducted a series of behavioral tests. Depressive‐like behavior was assessed using the tail suspension test (Kim et al. 2019). At 3 months postinjection (3 MPI) of α‐syn PFFs into the STR, mice did not exhibit significant differences compared to PBS‐injected controls in the open field test, pole test, rotarod performance, or grip strength (Figure 1A–F). However, a significant increase in immobility time during the tail suspension test was observed, indicating the presence of depressive‐like behavior (Figure 1G). At 6 months postinjection (6 MPI), PFF‐injected mice displayed marked motor impairments: Total distance traveled during the open field test were significantly reduced, the time to descend in the pole test was prolonged, latency to fall in the rotarod test was shortened, and grip strength was diminished (Figure 1C,E–G). Of note, PFF‐injected mice spent significantly less time in the center zone of the open field, indicating increased anxiety‐like behavior (Figure 1D) (Çevik et al. 2025). In addition, the reduction in total distance traveled may be associated with increased anxiety. Immobility time during the tail suspension test was further increased, suggesting worsening depressive‐like symptoms (Figure 1H). Based on the onset of motor deficits, we defined 3 MPI as the presymptomatic phase and 6 MPI as the symptomatic phase of the PD model. Notably, depressive‐like behavior was detectable prior to the onset of overt motor symptoms, aligning with clinical observations that mood disorders may precede motor dysfunction in a subset of PD patients (Assogna et al. 2020).

### 3.2 Progressive Degeneration of DNs and α‐Syn Pathology Following Striatal PFF Injection

**FIGURE 2:**
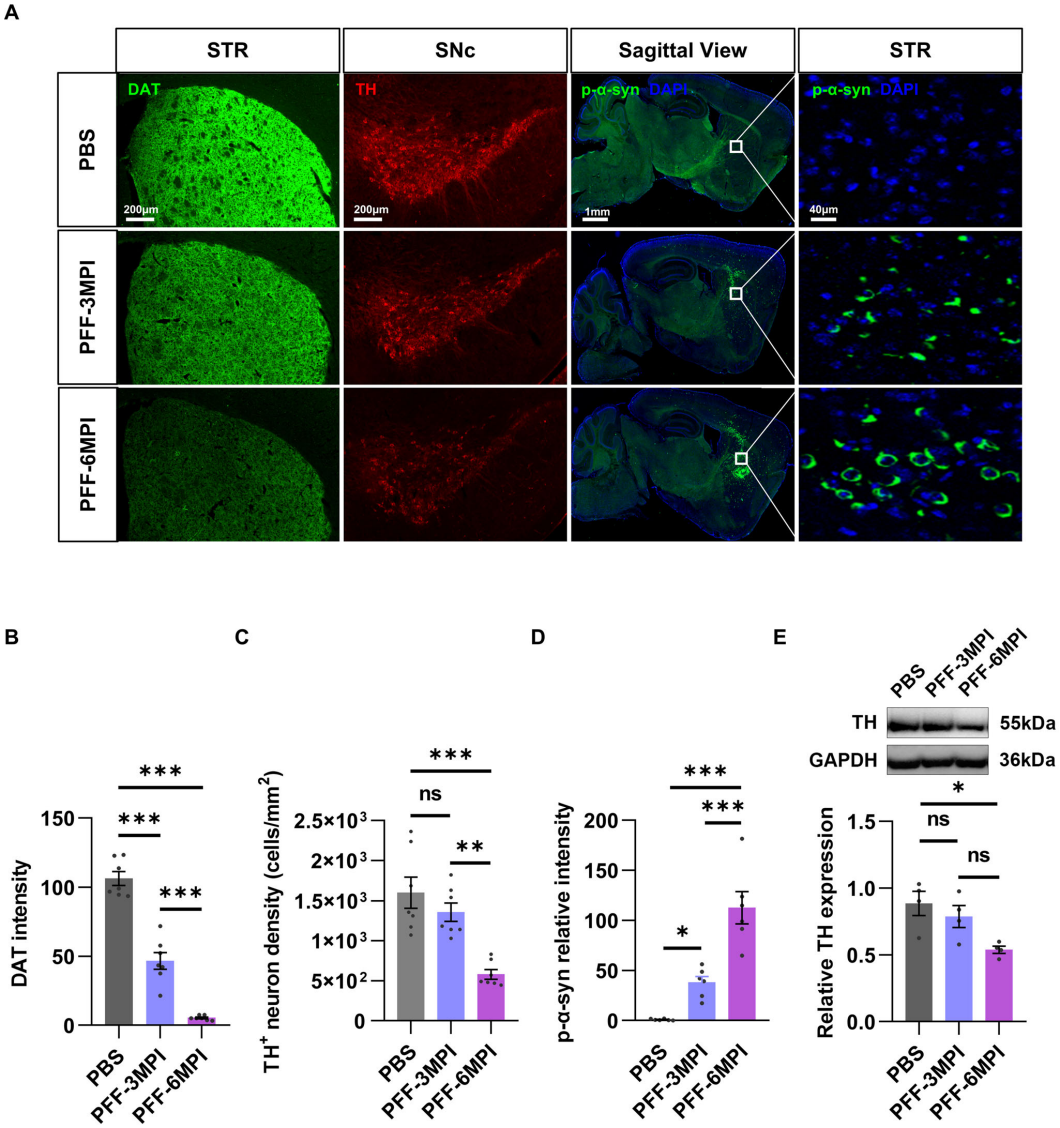
Progressive dopaminergic neurodegeneration and α‐synuclein pathology in the brains of α‐syn PFF‐injected mice. (A) Representative immunofluorescence images showing DAT expression in the striatum, TH‐positive neurons in the substantia nigra, and pS129 α‐synuclein in sagittal brain sections across groups. (B) Quantification of striatal DAT immunofluorescence intensity (PBS, *n* = 7; PFF‐3MPI, *n* = 7; PFF‐6MPI, *n* = 7). (C) Quantification of TH immunofluorescence in the substantia nigra (PBS, *n* = 7; PFF‐3MPI, *n* = 7; PFF‐6MPI, *n* = 7). (D) Quantification of pS129 α‐synuclein immunofluorescence intensity in sagittal brain sections (PBS, *n* = 6; PFF‐3MPI, *n* = 6; PFF‐6MPI, *n* = 6). (E) Representative Western blot of TH expression in the ventral midbrain and densitometric analysis (PBS, *n* = 4; PFF‐3MPI, *n* = 4; PFF‐6MPI, *n* = 4). Full‐length blots are presented in Figure S1. Data are presented as means ± SEM. *p < 0.05, **p < 0.01, ***p < 0.001; one‐way ANOVA. SNc, substantia nigra pars compacta; STR, striatum.

To assess dopaminergic neurodegeneration and pathological α‐syn accumulation, we performed immunofluorescence staining for DAT in the STR, TH‐positive neurons in the SN, and phosphorylated α‐syn at serine 129 (pS129, a marker of pathological α‐syn) in sagittal brain sections. Comparisons were made among PBS‐injected controls, PFF‐injected mice at 3 months (3 MPI), and 6 months (6 MPI). At 3 MPI, DAT expression in the STR was reduced in PFF‐injected mice compared to controls, and this reduction was more pronounced at 6 MPI (Figure 2A,B). Similarly, the density of TH‐positive neurons in the SN showed a time‐dependent decline, which became more pronounced at the 6‑month intervention time point. (Figure 2A,C). These findings were consistent with Western blot results showing a progressive decrease in TH protein levels in the ventral midbrain across the same time points (Figure 2E). Full Western blot images are provided in Figure S1A. In sagittal brain sections taken through the injection site, pS129 α‐syn immunoreactivity was detectable at 3 MPI, forming a prominent core around the injection locus. The intensity and spread of *p*‐α‐syn staining increased markedly by 6 MPI, indicating progressive accumulation and propagation of pathological α‐syn (Figure 2A,D). Collectively, striatal PFF injection in mice leads to progressive degeneration of dopaminergic terminals and cell bodies, accompanied by increasing levels of pathological α‐syn over a 6‐month period.

### 3.3 Visual Pathway Electrophysiological Impairment Emerges at 3 Months Post‐PFF Injection and Worsens by 6 Months Alongside Depth Perception Deficits

**FIGURE 3:**
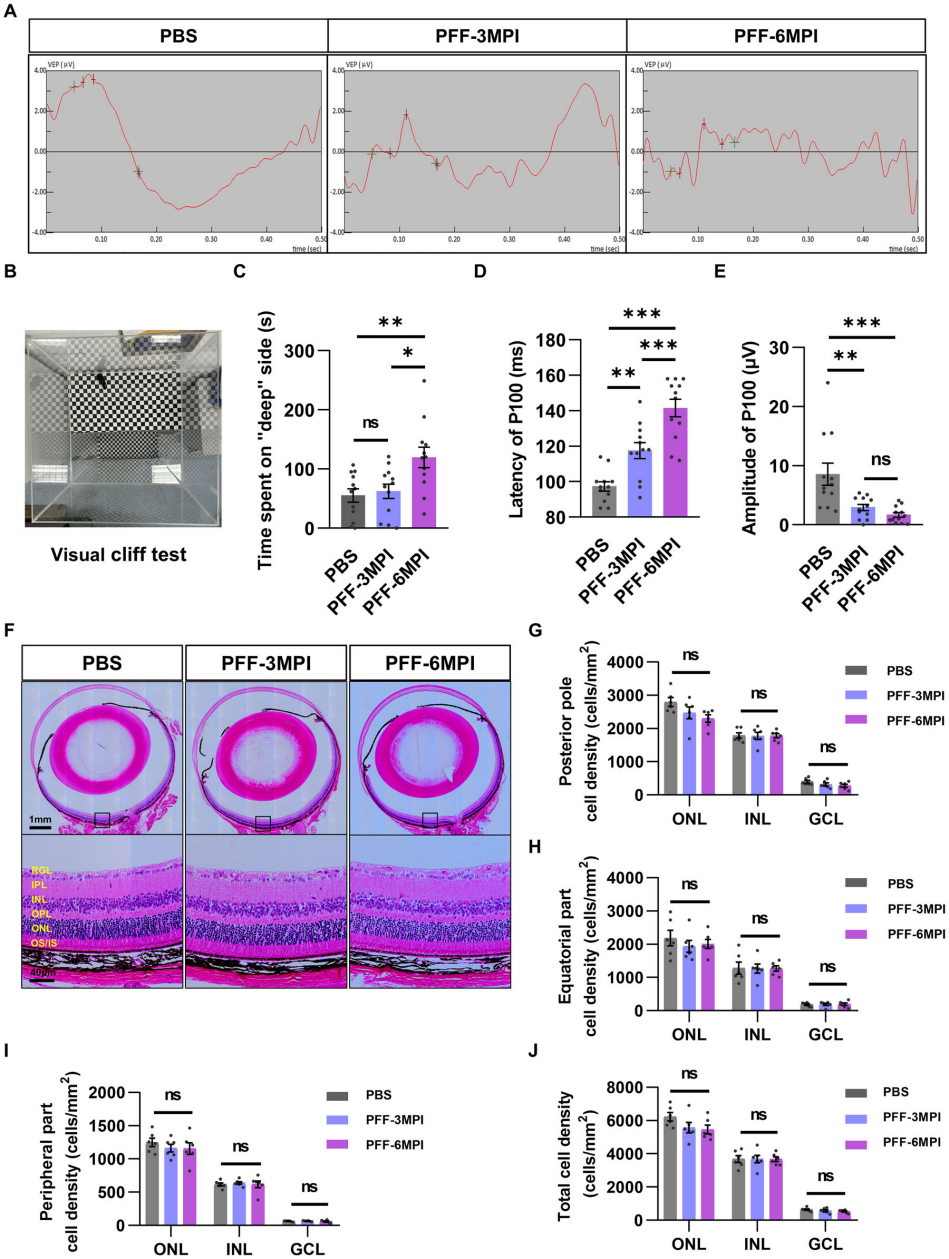
Visual pathway dysfunction precedes retinal structural loss in α‐syn PFF‐injected mice. (A) Representative PVEPs traces in control, 3 MPI, and 6 MPI mice. (B) Schematic diagram of the visual cliff test apparatus. (C) Quantification of time spent on the “deep” side of the platform across groups. (D) P100 latency comparison among groups. (E) P100 amplitude comparison among groups. Data are presented as means ± SEM. PBS, *n* = 12; PFF‐3MPI, *n* = 12; PFF‐6MPI, *n* = 12. *p < 0.05, **p < 0.01, ***p < 0.001; one‐way ANOVA. (F) Representative H&E‐stained retinal sagittal sections from each group. (G–I) Quantification of cell nuclei in GCL, INL, and ONL of the (G) posterior pole, (H) equator, and (I) peripheral regions. (J) Total nuclear counts across the entire retina. Data are presented as means ± SEM. PBS, *n* = 6; PFF‐3MPI, *n* = 6; PFF‐6MPI, *n* = 6. *p < 0.05, **p < 0.01, ***p < 0.001; one‐way ANOVA. GCL, ganglion cell layer; INL, inner nuclear layer; IPL, inner plexiform layer; ONL, outer nuclear layer; OPL, outer plexiform layer; PRL, photoreceptor layer.

To determine whether and when visual deficits emerge in the PFF mouse model, we evaluated visual function at 3 and 6 months postinjection (3 MPI and 6 MPI) using electrophysiological and behavioral assessments. Pattern‐reversal VEPs (PVEPs) were recorded in response to alternating black‐and‐white checkerboard stimuli, with P100 latency and amplitude used to assess the integrity of signal transmission from retinal ganglion cells (RGCs) to the VC (Batum et al. 2022; Tran et al. 2023). As early as 3 MPI, PFF‐injected mice exhibited a significant delay in P100 latency and reduction in amplitude, indicating functional impairment of the visual pathway. These abnormalities were exacerbated at 6 MPI (Figure 3A,D,E). Depth perception was assessed using the visual cliff test, in which a checkerboard pattern is positioned beneath a transparent platform to create an illusion of depth (Wang et al. 2023; Ji et al. 2022). At 3 MPI, PFF‐injected mice did not differ from controls in time spent on the “deep” side of the platform. However, at 6 MPI, they exhibited a significant reduction in depth preference, consistent with impaired depth perception (Figure 3B,C). These results indicate that although PFF‐injected mice do not exhibit overt visual behavioral deficits prior to the onset of motor symptoms, electrophysiological abnormalities and functional impairments in the visual pathway are already present.

To evaluate retinal structural integrity, we performed H&E staining on sagittal eye sections. Retinal cell bodies are primarily located in the GCL, INL, and ONL, which correspond to the somas of RGCs, bipolar/horizontal/amacrine/Müller cells, and photoreceptors, respectively (Nguyen‐Ba‐Charvet and Chédotal 2014). The retina was divided into three zones centered on the optic disc—posterior pole, equator, and peripheral regions—and cell nuclei were counted in the GCL, INL, and ONL of each region and across the whole retina. Quantitative analysis revealed no significant differences in cell density in any retinal layer or region between control, 3 MPI, and 6 MPI groups (Figure 3F–J), suggesting that retinal structure remains largely intact during the early stages of disease progression in this model. Collectively, these findings indicate that although retinal neurons are preserved morphologically in early‐stage PFF‐injected mice, functional deficits in the visual pathway are already apparent.

### 3.4 Pathological α‐Syn Accumulates in the Retina of α‐Syn PFF‐Injected Mice at 3 Months and Progressively Increases With Disease Severity

To characterize α‐syn pathology in the retina of the PD mouse model, we performed both Western blot and immunofluorescence staining to assess *p*‐α‐syn levels at 3 and 6 months postinjection (3 MPI and 6 MPI), compared to PBS‐injected controls.

**FIGURE 4:**
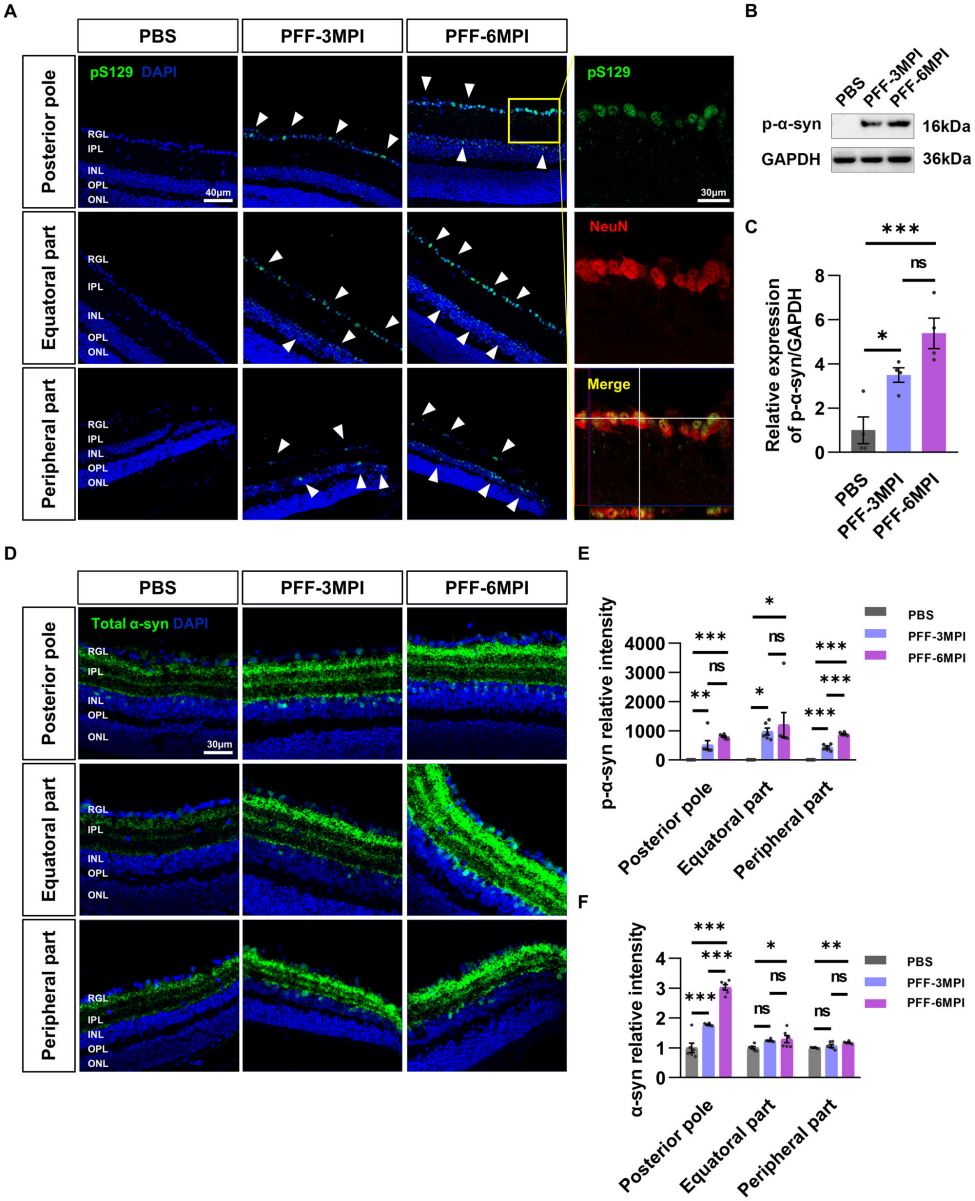
Retinal α‐synuclein pathology in α‐syn PFF‐injected mice. (A) Representative images showing *p*‐α‐synuclein immunofluorescence in the retina at different time points and co‐localization with NeuN. (B) Western blot of retinal *p*‐α‐synuclein expression in PBS, PFF‐3MPI, and PFF‐6MPI groups. Full‐length blots are presented in Figure S1. (C) Quantification of *p*‐α‐synuclein protein levels based on band intensity. (D) Representative images of total α‐synuclein immunofluorescence in the retina across groups. (E) Quantification of *p*‐α‐synuclein fluorescence intensity. (F) Quantification of total α‐synuclein fluorescence intensity. Data are presented as means ± SEM. Immunofluorescence quantification: PBS, *n* = 6; PFF‐3MPI, *n* = 6; PFF‐6MPI, *n* = 6. Western blot quantification: PBS, *n* = 4; PFF‐3MPI, *n* = 4; PFF‐6MPI, *n* = 4. *p < 0.05, **p < 0.01, ***p < 0.001; one‐way ANOVA. GCL, ganglion cell layer; INL, inner nuclear layer; IPL, inner plexiform layer; ONL, outer nuclear layer; OPL, outer plexiform layer.

Western blot analysis demonstrated detectable expression of *p*‐α‐syn in retinal lysates at 3 MPI, with a marked increase in expression at 6 MPI (Figure 4B,C). Full Western blot images are provided in Figure S1B. Immunofluorescence staining revealed *p*‐α‐syn‐positive inclusions in the GCL at 3 MPI, with *p*‐α‐syn‐positive puncta also observed in the INL and inner plexiform layer (IPL). These features became more prominent by 6 MPI, with increased fluorescence intensity and more widespread distribution (Figure 4A,E). In the RGC layer, pSer129‑positive signals were observed to colocalize with NeuN‑labeled neuronal nuclei, suggesting possible nuclear or perinuclear accumulation of p‑α‑syn pathology (Figure 4A).

Immunofluorescence staining for total α‐syn showed its primary localization in the GCL, IPL, and INL. Compared with control mice, total α‐syn fluorescence intensity was increased at 3 MPI and became more pronounced at 6 MPI (Figure 4D,F).

### 3.5 The Expression of TH Is Reduced in the Retina of α‐Syn PFF‐Injected Mice

To investigate whether retinal TH expression and TH‐positive amacrine cells are affected in the α‐syn PFF model, we performed Western blot and immunofluorescence analyses of the retina at 3 and 6 months postinjection.

**FIGURE 5:**
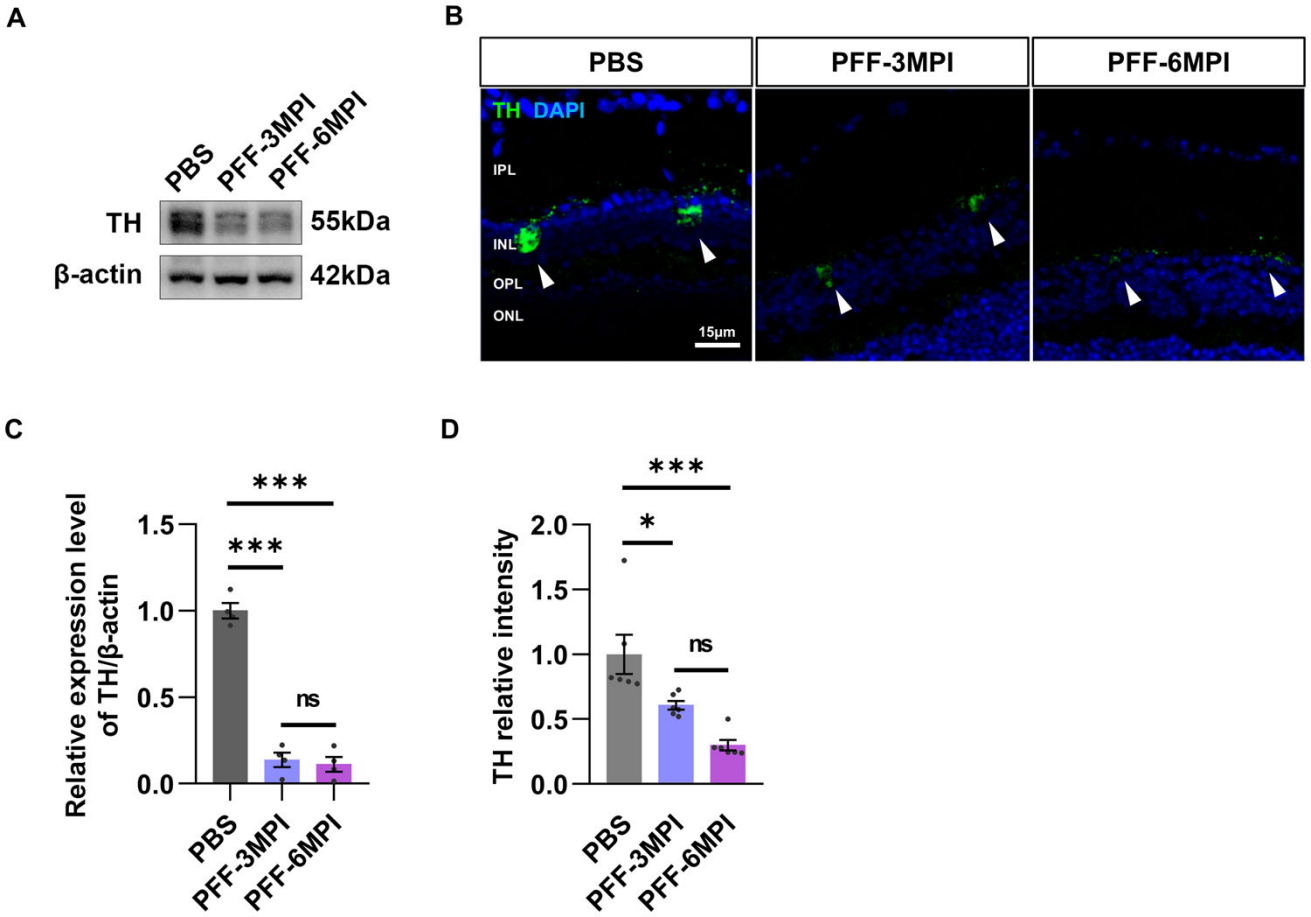
Decreased retinal tyrosine hydroxylase (TH) expression in α‐syn PFF‐injected mice. (A) Representative Western blot images of TH expression in the retinas of PBS, PFF‐3MPI, and PFF‐6MPI mice. Full‐length blots are presented in Figure S1. (B) Representative immunofluorescence images of TH‐positive dopaminergic amacrine cells in each group. (C) Quantification of TH protein levels by densitometry. (D) Quantification of TH immunofluorescence intensity in the retina. Data are presented as means ± SEM. Western blot quantification: PBS, *n* = 4; PFF‐3MPI, *n* = 4; PFF‐6MPI, *n* = 4. Immunofluorescence quantification: PBS, *n* = 6; PFF‐3MPI, *n* = 6; PFF‐6MPI, *n* = 6. *p < 0.05, **p < 0.01, ***p < 0.001; one‐way ANOVA. INL, inner nuclear layer; IPL, inner plexiform layer; ONL, outer nuclear layer; OPL, outer plexiform layer.

Western blot results revealed a progressive decrease in TH protein expression in the retina of PFF‐injected mice compared to PBS controls (Figure 5A,C). Full Western blot images are provided in Figure S1C. Immunofluorescence staining demonstrated that TH‐positive amacrine cells were dendritic arbors extended toward the boundary between the INL and the IPL. At 3 MPI, a reduction in TH immunoreactivity was evident, which became more pronounced by 6 MPI (Figure 5B,D), in agreement with the Western blot data.

### 3.6 Microglial Activation and Transient Müller Glial Reactivity in the Retina of α‐Syn PFF‐Injected Mice

**FIGURE 6:**
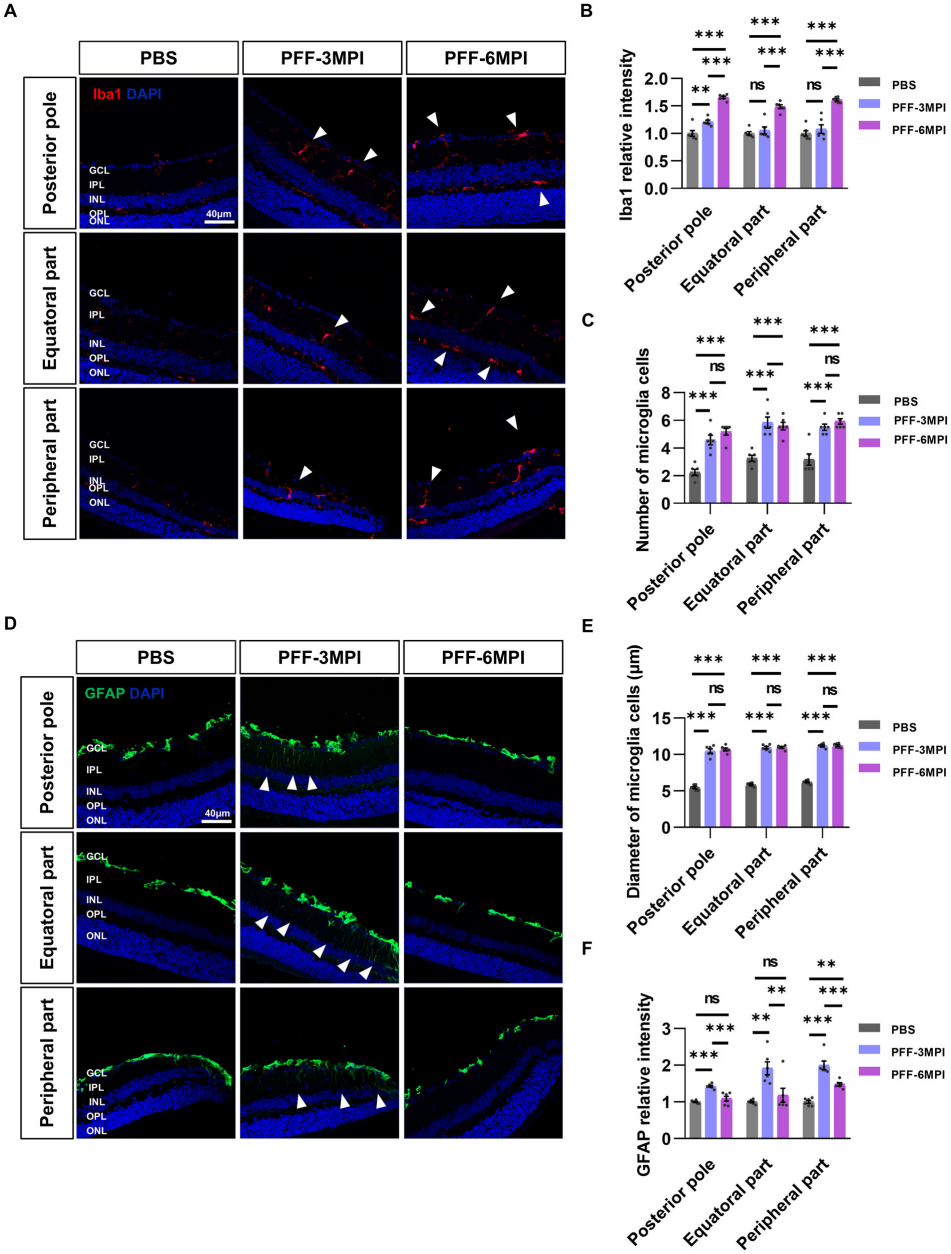
Microglial activation and transient Müller glial reactivity in the retinas of α‐syn PFF‐injected mice. (A) Representative Iba1 immunofluorescence images showing microglial distribution in different retinal layers. (B) Quantification of Iba1 fluorescence intensity across groups. (C) Quantification of microglial cell counts in each group. (D) Representative GFAP immunofluorescence images showing Müller cell reactivity in different retinal regions. (E) Quantification of microglial soma diameter across groups. (F) Quantification of GFAP fluorescence intensity in the retina. Data are presented as means ± SEM. Group sizes: PBS, *n* = 6; PFF‐3MPI, *n* = 6; PFF‐6MPI, *n* = 6. *p < 0.05, **p < 0.01, ***p < 0.001; one‐way ANOVA. GCL, ganglion cell layer; INL, inner nuclear layer; IPL, inner plexiform layer; ONL, outer nuclear layer; OPL, outer plexiform layer.

To investigate microglial changes in the retina of PD model mice, we performed Iba1 immunofluorescence staining. Microglia were primarily distributed in the GCL, IPL, INL, and outer plexiform layer (OPL). At 3 months postinjection (3 MPI), microglia exhibited clear signs of activation, including soma enlargement and increased cell number across retinal layers. This reactive profile persisted at 6 months postinjection (6 MPI), accompanied by a progressive increase in Iba1 fluorescence intensity (Figure 6A–C,E), suggesting that microglial proliferation and activation may contribute to retinal pathology in the PFF model.

Müller cells are a specialized type of retinal glial cell that provide metabolic and structural support to neurons, regulate the extracellular environment, and play protective and regenerative roles under pathological conditions (Thiel et al. 2022). Glial fibrillary acidic protein (GFAP), a canonical marker of astrocytes in the brain, is also expressed in retinal astrocytes and, under stress or injury, in activated Müller cells—particularly in their endfeet. Under physiological conditions, GFAP is minimally expressed in Müller cells, but pathological stimuli induce its upregulation, making GFAP a reliable indicator of Müller cell gliosis. GFAP immunofluorescence staining showed a transient activation of Müller glia in the retina of PFF‐injected mice. At 3 MPI, GFAP expression in the GCL was markedly increased, with enhanced staining in the processes extending into the IPL and INL (Figure 6D,F). However, by 6 MPI, GFAP levels returned to near control levels, suggesting an early, transient gliotic response of Müller cells following retinal stress in the PFF model.

### 3.7 α‐Syn Pathology Is Detected in the VC at 3 Months Post‐PFF Injection

**FIGURE 7:**
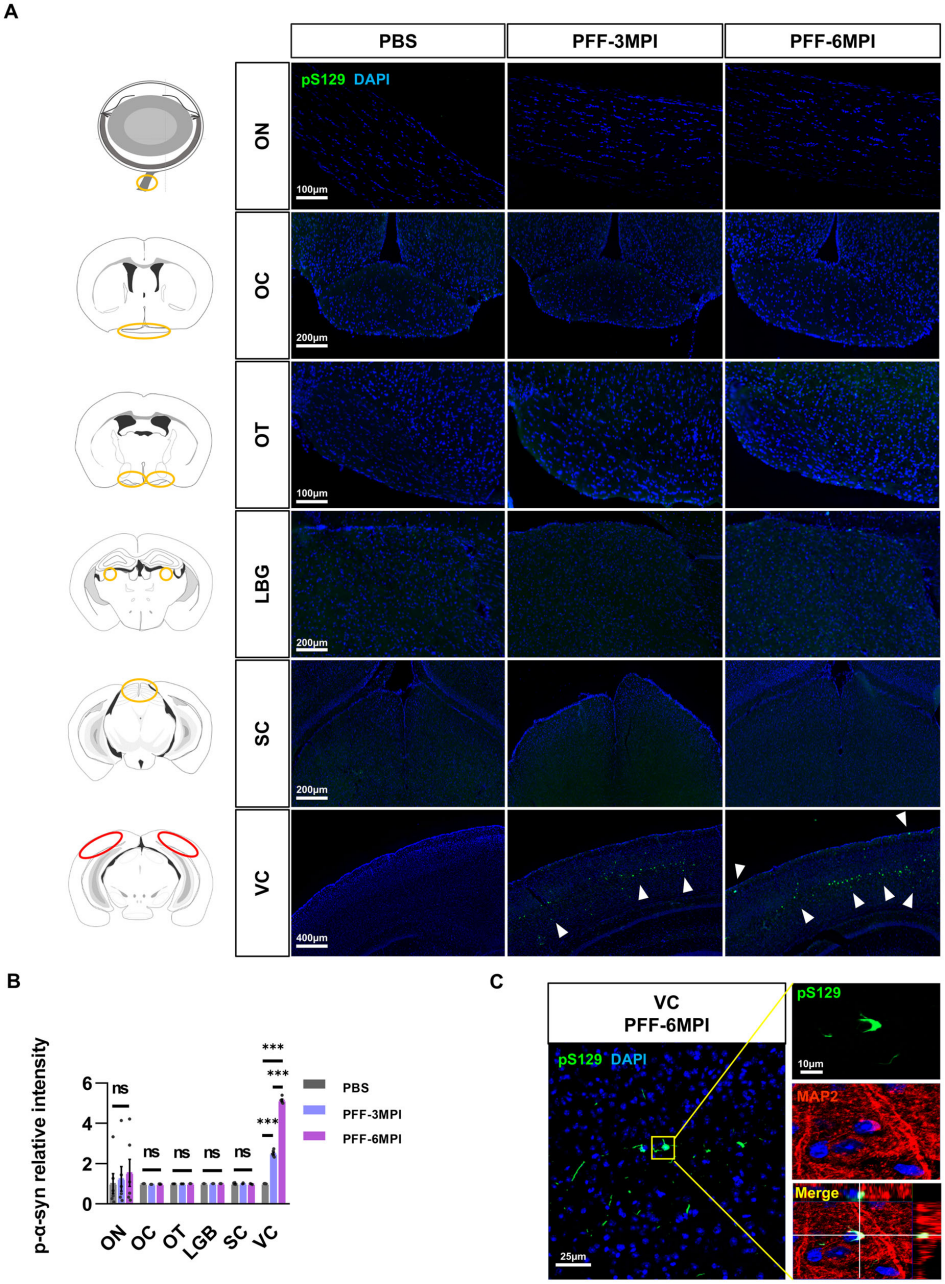
Expression of *p*‐α‐synuclein in the visual cortex of α‐synuclein PFF‐injected mice. (A) Representative images of *p*‐α‐synuclein immunofluorescence in the optic nerve, optic chiasm, optic tract, lateral geniculate nucleus, superior colliculus, and visual cortex across groups. (B) Quantification of *p*‐α‐synuclein immunofluorescence intensity in the visual pathway (PBS, *n* = 6; PFF‐3MPI, *n* = 6; PFF‐6MPI, *n* = 6). (C) Representative image showing co‐localization of *p*‐α‐synuclein with MAP2 in the visual cortex. Data are presented as means ± SEM. *p < 0.05, **p < 0.01, ***p < 0.001; one‐way ANOVA. LGN, lateral geniculate nucleus; OC, optic chiasma; ON, optic nerve; OT, optic tract; VC, visual cortex.

To determine whether synucleinopathy extends beyond the retina in our PFF‐induced PD mouse model, we examined *p*‐α‐syn accumulation via immunofluorescence staining in multiple components of the visual pathway, including the ON, OC, OT, LGN, superior colliculus, and VC. Surprisingly, *p*‐α‐syn immunoreactivity was only detected in the VC of PFF‐injected mice. At 3 months postinjection, the *p*‐α‐syn‐positive signal was already evident in the VC and became more pronounced at 6 months (Figure 7A,B). Co‐immunostaining with MAP2, a neuronal marker, showed clear co‐localization with *p*‐α‐syn, indicating that pathological α‐syn accumulates within neuronal somata in the VC (Figure 7C).

### 3.8 Pathological *p*‐α‐Syn Accumulates in Visual Association Cortices of PFF‐Injected Mice

**FIGURE 8:**
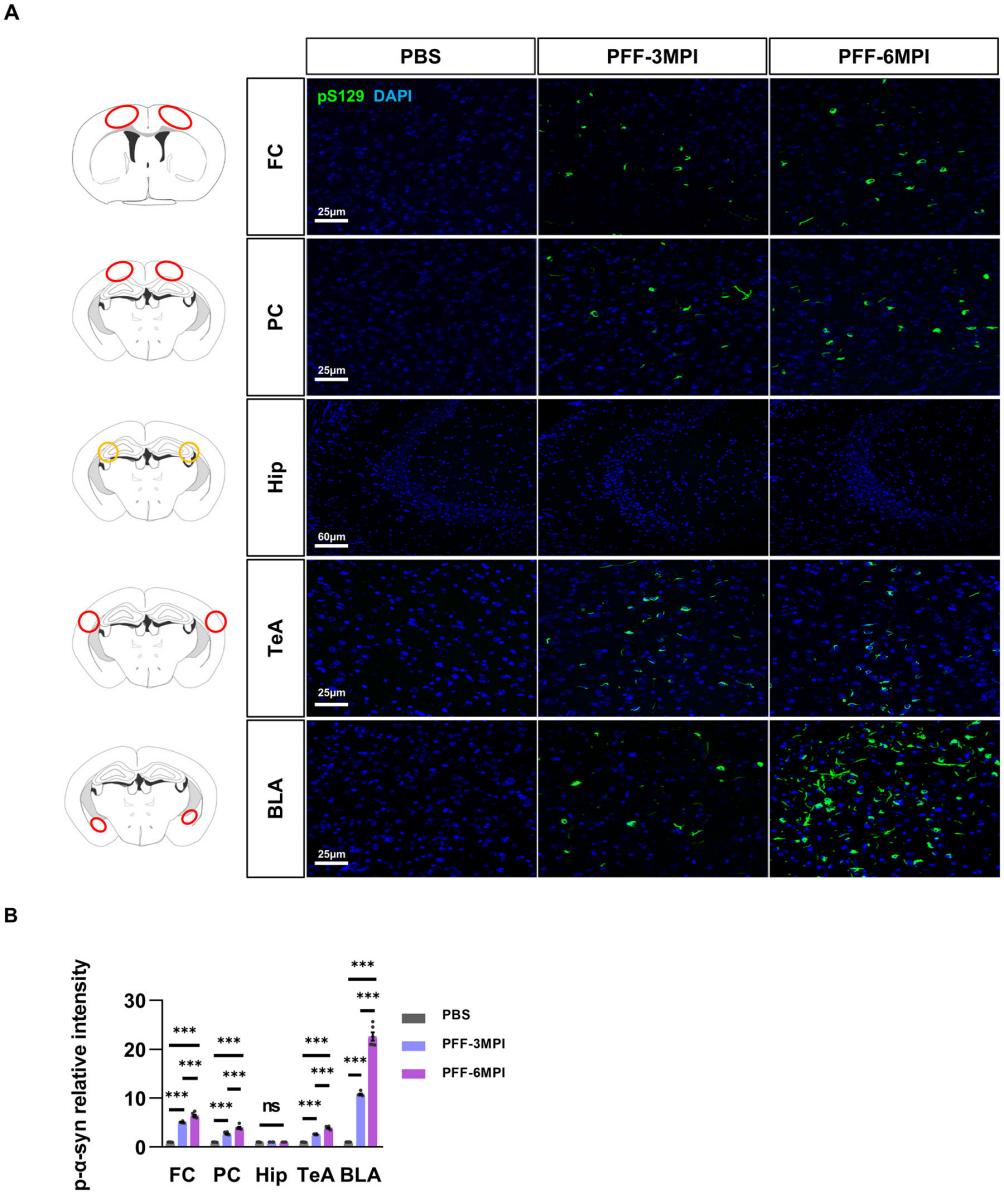
*p*‐α‐Synuclein accumulation in frontal, parietal, and temporal association cortices, as well as the amygdala, in PFF‐injected mice. (A) Representative immunofluorescence images showing *p*‐α‐synuclein staining in the frontal cortex, parietal cortex, hippocampus, temporal association cortex, and amygdala across experimental groups. (B) Quantification of *p*‐α‐synuclein fluorescence intensity in each region (PBS, *n* = 6; PFF‐3MPI, *n* = 6; PFF‐6MPI, *n* = 6). Data are presented as means ± SEM. *p < 0.05, **p < 0.01, ***p < 0.001; one‐way ANOVA. BLA, basolateral nucleus of amygdale; FC, frontal motor cortex; Hip, hippocampus; PC, parietal cortex; TeA, temporal association cortex.

To assess whether synucleinopathy extends into the PC, frontal cortex, hippocampus (Hip), TeA, and amygdala in PFF‐injected mice, immunofluorescence staining for *p*‐α‐syn was used. At 3 months postinjection (3 MPI), *p*‐α‐syn immunoreactivity was already detectable in the frontal, parietal, and temporal association cortices, as well as in the amygdala. These signals intensified significantly by 6 months postinjection (6 MPI), as indicated by quantitative analysis of fluorescence intensity (Figure 8A,B).

## 4 Discussion

In this study, we established a sporadic PD model in wild‐type C57BL/6J mice by intrastriatal injection of α‐syn PFFs, which mimics nontoxin‐induced forms of idiopathic PD. We comprehensively assessed visual function, visual electrophysiology, retinal pathology, and pathological α‐syn deposition along the visual pathway at both presymptomatic (3 months postinjection) and symptomatic (6 months postinjection) stages. Our findings revealed that functional impairments of the visual system emerge prior to the onset of motor deficits, such as altered pattern‐reversal VEPs (PVEPs), characterized by prolonged P100 latency and reduced amplitude. These electrophysiological abnormalities were accompanied by early pathological changes in the retina, including *p*‐α‐syn accumulation, increased total α‐syn levels, decreased TH expression, and microglial activation. Notably, these changes became more pronounced at 6 months postinjection, whereas no significant loss of retinal neurons was observed in any layer up to this time point. Within the primary visual pathway, in addition to the retina, *p*‐α‐syn immunoreactivity was detected in the VC as early as 3 months postinjection. Interestingly, no pathological α‐syn accumulation was observed in the ON, OC, OT, LGN, or superior colliculus. Furthermore, *p*‐α‐syn deposition was also observed at 3 months in visual association areas involved in higher‐order visual processing, including the frontal, parietal, and temporal association cortices, as well as the amygdala.

Visual impairment represents a prevalent nonmotor manifestation of PD, negatively impacting essential daily activities and contributing to reduced self‐efficacy and overall quality of life (Weil et al. 2016; Hamedani et al. 2020). In recent years, increasing attention has been directed toward structural and functional alterations in the visual pathway in PD. A prospective longitudinal study by Hasanov et al.^2019^ involving 19 matched subjects demonstrated significantly prolonged latency and reduced amplitude of the P100 wave in early‐stage PD patients. In a cross‐sectional study of 59 PD patients, Sipos‐Lascu et al. (2024) validated a correlation between VEP P100 latency and the severity of motor symptoms. Aras et al. (2014) observed prolonged VEP latencies in an MPTP‐induced mouse model of PD. While the model showed marked loss of DNs in the SN, increased caspase‐3 activity, nitrite/nitrate levels, and 4‐hydroxynonenal (4‐HNE) accumulation, these pathological changes were not detected in the retina. Treatment with 7‐nitroindazole (7‐NI) and *S*‐methylisothiourea (SMT) partially reversed these effects, suggesting that VEP abnormalities may reflect upstream dopaminergic degeneration and oxidative stress‐induced lipid peroxidation Similarly, Østergaard et al. (2020) reported delayed VEP latencies in the late stages of disease in rats receiving unilateral intranigral AAV‐mediated α‐syn overexpression. Notably, human α‐syn was detected in the ipsilateral STR and superior colliculus, with VEP changes occurring later than protein deposition. This delay may be attributed to downstream effects, such as posttranslational modifications (e.g., phosphorylation or aggregation) of α‐syn Due to practical and ethical constraints, assessing VEP changes during the prodromal phase of PD in clinical populations remains challenging, and the utility of VEPs for early diagnosis is still unclear. Animal models offer a valuable platform to investigate early electrophysiological and pathological changes in the visual system. The α‐syn PFF model faithfully reproduces key pathological features of PD and offers distinct advantages in modeling chronic, idiopathic‐like progression and prion‐like propagation of α‐syn. In our current study, we applied the intrastriatal α‐syn PFF injection model to explore both electrophysiological and pathological alterations in the visual system. By using 3‐ and 6‐month postinjection time points, we aimed to represent the prodromal and symptomatic stages of PD, respectively, thus providing an optimal framework to study early visual dysfunction. Our findings revealed that subclinical changes in the visual pathway occur as early as the prodromal stage, with VEPs serving as a sensitive tool to detect these early abnormalities. Furthermore, VEP alterations were more pronounced during the symptomatic stage, consistent with previous clinical studies suggesting VEP parameters correlate with disease progression. Collectively, our findings highlight that VEPs may serve as a sensitive tool for identifying early visual deficits associated with PD. Nonetheless, additional research is required to validate their diagnostic specificity and explore their applicability in clinical settings.

Aberrant accumulation of α‐syn is a defining pathological feature of PD, contributing to the progressive degeneration of DNs in the SNc. Beyond the central nervous system, α‐syn pathology has also been identified in the retina of PD patients. Bodis‐Wollner et al. (2014) were the first to report postmortem retinal α‐syn alterations, including intracellular Lewy body (LB)‐like aggregates in the INL, diffuse α‐syn deposition and Lewy neurites in the IPL, as well as cytoplasmic α‐syn‐positive inclusions within the GCL (Armstrong 2017). Subsequently, Beach et al. (2014) used immunohistochemical staining to detect *p*‐α‐syn in the RNFL, GCL, and the inner IPL. Current studies consistently suggest that RGCs and DACs are particularly vulnerable to synucleinopathy in PD (Beach et al. 2014; Ortuño‐Lizarán et al. 2018; Bodis‐Wollner et al. 2014; Ho et al. 2014). Among classical neurotoxin‐induced PD models, only the rotenone‐induced rat model reported by Normando et al. (2016) showed diffuse α‐syn immunoreactivity in the retina. In transgenic models, Veys et al. (2019) demonstrated *p*‐α‐syn immunopositivity in the cell bodies of GCL and INL (adjacent to the IPL), as well as in dendrites within the IPL and RNFL of (Thy‐1) h[A30P] α‐syn transgenic mice. However, differences in the spatial pattern of retinal *p*‐α‐syn expression have been reported across various models. For example, Mammadova et al. (2019) accelerated retinal α‐syn pathology by intracerebral inoculation of brain homogenates from aged TgM83 mice into young (2‐month‐old) TgM83 recipients. They found a significant increase in retinal α‐syn at 8 months of age compared to non‐inoculated controls, with pSer129‐positive immunoreactivity observed in photoreceptor outer segments, ONL, OPL, and INL (Beach et al. 2014). Similarly, Tran et al. (2023) reported pSer129 expression in the ONL, OPL, and photoreceptor layers in A53T homozygous α‐syn transgenic mice compared to wild‐type controls. In our model, *p*‐α‐syn immunoreactivity was already detectable in the retina during the prodromal phase of PD. pSer129‑positive signals were observed to colocalize with NeuN‑labeled neurons in the retinal GCL (Figure 4A). NeuN is a well‑established marker of mature neurons, with predominant expression in the nucleus and additional localization in the perinuclear cytoplasm. Therefore, the colocalization of pSer129 with NeuN suggests that these signals are associated with the nuclear or perinuclear compartment of RGCs, although the precise subcellular localization cannot be determined solely by conventional confocal microscopy. Nuclear localization of α‑syn has been reported in animal models of PD and in postmortem brain tissue from PD patients (Mason et al. 2016; Mason et al. 2019; Horan‐Portelance et al. 2026; Ermini et al. 2024). Perinuclear accumulation of pSer129 is thought to reflect dynein‑dependent transport of misfolded proteins to the aggresome formation site near the microtubule organizing center. The functional significance of this perinuclear localization remains unclear, but it may represent an early stage of inclusion formation or a cellular response to proteostatic stress (Dinter et al. 2020; Mansuri et al. 2024). Although nuclear localization of pSer129 has been reported in certain experimental settings (Koss et al. 2022; Millett et al. 2025), we interpret our finding cautiously. Direct evidence of intranuclear pSer129 would require additional high‑resolution imaging with orthogonal views or co‑staining with nuclear markers such as DAPI or Hoechst, which were not available for this specific image. Therefore, we describe our observation as perinuclear or perinuclear‑associated accumulation of pSer129, and note that future studies using higher‑resolution confocal microscopy with appropriate nuclear counterstaining will be necessary to clarify the exact subcellular distribution of pSer129 in retinal neurons. In addition, punctate *p*‐α‐syn staining was observed in the cytoplasm of certain INL cells and throughout the IPL, and these changes became more pronounced following the onset of motor symptoms. Moreover, total α‐syn levels were significantly increased in the GCL, INL, and IPL layers in PFF‐injected mice compared to controls, suggesting that the “seeding” activity of exogenous PFFs may have induced pathological accumulation of endogenous α‐syn. These findings highlight the high vulnerability of RGCs to α‐synucleinopathy. As the axons of RGCs converge to form the ON, their integrity is essential for the transmission of visual signals. The INL comprises somas of DACs, bipolar cells, horizontal cells, and Müller glia, while the IPL is composed of synaptic terminals and dendritic processes from these neurons and RGCs. These layers are involved in visual signal integration, neuroregeneration, and immunomodulation (Østergaard et al. 2020). The accumulation of *p*‐α‐syn can lead to cytotoxic effects such as the formation of Lewy bodies, impairment of proteasomal and autophagic pathways, disruption of intracellular transport, and mitochondrial dysfunction, ultimately resulting in neuronal dysfunction or death (Beach et al. 2014; Ho et al. 2014). The retinal distribution of *p*‐α‐syn in our PFF model is consistent with several key pathological studies in PD patient retinas (Volpicelli‐Daley et al. 2014; Beach et al. 2014), supporting its validity as a representative model of retinal synucleinopathy. The observed differences in *p*‐α‐syn localization across animal models may reflect distinct pathogenic mechanisms related to genetic backgrounds, as many of the aforementioned models involve transgenic expression of mutant α‐syn.

Ortuno‐Lizaràn et al. (2018) reported a positive correlation between the density of retinal and cerebral *p*‐α‐syn in PD patients, which also correlated with motor severity and disease stage, suggesting that the retina may serve as a potential biomarker for PD (Beach et al. 2014). In our study, we observed not only a progressive increase in retinal *p*‐α‐syn expression with disease progression but also its presence during the premotor phase. These findings indicate that pathological α‐syn deposition in the retina may be a valuable marker not only for tracking disease progression but also for early diagnosis. Thus, future studies exploring noninvasive in vivo visualization of retinal pathological α‐syn are of considerable clinical interest. For example, retinal imaging using fluorescently labeled *p*‐α‐syn antibody‐conjugated contrast agents could represent a promising strategy, though its feasibility remains to be validated through further research.

In addition to α‐syn pathology, we also detected other retinal changes analogous to hallmark brain pathology in PD, such as reduced TH expression and microglial activation. Notably, these changes were already present before motor deficits appeared and became more pronounced as the disease progressed. These observations underscore the retina's promise as a noninvasive and readily accessible site for early‐stage PD detection and longitudinal monitoring. Owing to its embryological origin from the neuroectoderm, the retina is considered a peripheral extension of the central nervous system and is often described as a “window to the brain.” Consistent with this view, disease‐specific retinal abnormalities have been documented in PD, Alzheimer's disease (AD), and other neurodegenerative conditions (Guo et al. 2018). In our model, significant pSer129‐positive signals were observed in the GCL, IPL, and INL. pSer129 pathology are known to activate microglia and trigger a chronic inflammatory response, which can lead to neuronal dysfunction and death. Persistent inflammation may also impair the autophagic and phagocytic functions of microglia, further exacerbating α‐syn pathology (Scheiblich et al. 2021; Lv et al. 2023; Wang et al. 2021). In the MPTP‐induced PD model, retinal DACs were reduced, accompanied by decreased retinal DA levels and slowed DA‐dependent feedback circuitry (Tran et al. 2022; Huang and Yin 2011), but no synuclein pathology was reported. Although some transgenic models, such as the Plp‐α‐syn mouse (Kaehler et al. 2020), Thy1‐h[A30P] α‐syn mouse (Veys et al. 2021), and TgM83 inoculated model (Mammadova et al. 2019), have demonstrated α‐syn accumulation and degeneration of pigmented RGCs and DACs, the localization of *p*‐α‐syn often differs from that seen in PD patients. This limits their ability to replicate sporadic PD without genetic mutations. Recently, Pérez‐Acuña et al. (2023)showed that 2 months after intravitreal injection of mouse‐derived α‐syn PFFs, peripheral TH expression in the retina was reduced. They also observed retinal *p*‐α‐syn aggregation, DN loss, and retinal thinning; however, they did not assess visual function in this model. Taken together, our model not only recapitulates key features of central PD pathology in the retina but also enables functional and pathological monitoring during the prodromal stage. Importantly, we did not observe significant retinal neuronal loss within the first 6 months. Only subtle trends toward cell number reduction were noted in the posterior GCL and ONL, regions populated by ganglion and photoreceptor cell bodies. This suggests that overt retinal cell loss may occur during the middle to late stages of disease in this model. Moreover, TH‐positive amacrine cells represent only a small proportion of the INL, making early cell loss in this layer more difficult to detect.

Furthermore, in our model, transient activation of Müller glial cells was observed in the retina at 3 months post‐PFF injection. Müller cells are the principal type of macroglial cells in the retina and interact with nearly all types of retinal neurons, playing essential metabolic and supportive roles. They regulate neuronal excitability by uptaking and recycling neurotransmitters and by releasing neuroactive substances. In various models of photoreceptor or RGC injury, transplanted Müller glia have been shown to secrete neuroprotective factors that facilitate the repair and survival of damaged neurons, thereby partially restoring visual function (Eastlake et al. 2021). Under appropriate stimuli, Müller cells may undergo dedifferentiation and exhibit the capacity to transdifferentiate into other neuronal phenotypes, including dopaminergic‐like cells (da Silva et al. 2019). These cells are capable of synthesizing DA and releasing it into the extracellular matrix. In a mouse model involving the transplantation of striatal Müller glia, DA supplementation by these cells was observed, suggesting their potential to ameliorate dopaminergic deficits (Stutz et al. 2014). Although the specificity of Müller glial activation and proliferation in the context of PD remains to be fully elucidated, their neuroregenerative potential offers a promising avenue for alternative therapeutic strategies (Zhou et al. 2020).

In addition to the retina, other components of the visual pathway and visual association cortices also contribute to the perception, transmission, and processing of visual information. The visual pathway serves as the fundamental neural basis for visual information transfer. After initial processing in the retina, visual signals are transmitted via the ON. Nasal retinal fibers cross at the OC, where they merge with uncrossed temporal fibers to form the OT. Most of these fibers project to the LGN and subsequently to the VC, completing the primary visual transmission. A smaller subset of fibers project to the superior colliculus and pretectal area, which are involved in the pupillary light reflex (Heermann 2017). Visual information processed in the primary VC is further integrated and distributed along two parallel streams: the ventral stream and the dorsal stream. The ventral stream is primarily involved in object recognition and spatial perception and includes regions such as the Hip and amygdala. The dorsal stream processes motion and spatial localization and encompasses areas such as the temporoparietal association cortex, parietal lobe, and frontal lobe (Li et al. 2023). Lesions in these cortical regions or disruptions in their network connectivity have been associated with higher‐order visual disturbances, such as visuospatial deficits and visual hallucinations (Volpicelli‐Daley et al. 2014; Nishio et al. 2018). Recent neuroimaging studies using functional MRI and 18F‐fluorodeoxyglucose positron emission tomography (FDG‐PET) in PD patients have revealed both structural and functional alterations not only in the primary visual pathway—including the VC, optic radiations, LGN, and OC—but also within the broader visual association cortices (Li et al. 2023; Haug et al. 1994; Arrigo et al. 2017). Moreover, functional connectivity abnormalities have been documented among key visual association networks(Baik et al. 2024; Nishio et al. 2018; Yao et al. 2014; Liu et al. 2023; Marques et al. 2022; Erramuzpe et al. 2025), suggesting that PD‐related visual impairment is multifactorial. In our study, we detected *p*‐α‐syn immunoreactivity in the VC, parietal lobe, frontal lobe, TeA, and amygdala as early as 3 months post‐PFF injection. This immunoreactivity was markedly increased at 6 months, coinciding with the trend of VEP alterations. Notably, abnormalities in visual depth perception and motor deficits were only observed at 6 months. These findings suggest that both functional and pathological alterations in the visual pathway precede the onset of overt visual symptoms and motor dysfunction. Collectively, our results support the hypothesis that retinal and central visual structures may cooperatively contribute to the development of visual deficits in PD.

In our study, striatal injection of α‐syn PFFs successfully induced *p*‐α‐syn accumulation in the STR, cerebral cortex, and amygdala. Notably, early pathological changes were also observed in the retina, including increased expression of *p*‐α‐syn and elevated total α‐syn levels, suggesting the possibility of pathological α‐syn propagation from the brain to the retina. α‐Syn aggregates are known to spread via multiple mechanisms, including prion‐like transmission (Beach et al. 2014; Irwin et al. 2013; Song and Li 2025), tunneling nanotube‐mediated transport (Chakraborty et al. 2023; Duan et al. 2023), and exosome‐mediated transfer (Xia et al. 2019; Guo et al. 2020). The anatomical proximity between the STR and the frontal/parietal cortices, as well as their extensive neural connectivity with cortical and limbic structures, such as the amygdala (Choi et al. 2017; Lee et al. 2024), may facilitate α‐syn propagation through neural projections or prion‐like mechanisms. However, the current study did not directly investigate the mechanistic pathways underlying the spread of α‐syn pathology from the brain to the retina, which warrants further investigation. Cao et al. (2024) recently employed immunofluorescent 3D imaging and cisterna magna‐based stereotaxic tracer delivery to explore the transmission of β‐amyloid (Aβ) from the brain to the eye in AD patients and 5×FAD mice. They identified three primary brain‐to‐eye Aβ transport routes: the ON sheath‐lymphatic pathway, the interaxonal space pathway, and the perivascular space along arteries. Moreover, they demonstrated that aquaporin‐4 (AQP4) regulates both brain‐to‐eye transport and retinal glymphatic clearance of Aβ, thereby modulating ocular Aβ deposition and associated pathology (Cao et al. 2024). In our PFF‐induced PD mouse model, retinal α‐syn pathology was evident as early as 3 months postinjection. By 6 months, while pathological α‐syn accumulation was observed in the VC, no immunopositive signals for *p*‐α‐syn were detected in other primary components of the visual pathway, such as the ON, OC, OT, LGN, or superior colliculus. This limits the evidence for retrograde α‐syn propagation along the visual pathway. Whether humoral transmission contributes to the dissemination of pathology to the retina remains uncertain and merits future exploration. Understanding the route of α‐syn propagation from the brain to the retina may offer novel insights into the pathogenesis of nonmotor symptoms in PD and reveal potential therapeutic targets.

Several limitations of this study should be acknowledged. First, behavioral tests such as the open field and depth perception task are sensitive to anxiety‐like states, and the observed reductions in center zone exploration and total distance traveled may partially reflect increased anxiety rather than pure motor or sensory deficits. Nevertheless, the primary finding of this study remains unaffected: Significant PVEP abnormalities and retinal pathological changes were already evident at 3 months postinjection, whereas no behavioral deficits other than depressive‐like behavior were detected at this time point. This temporal dissociation indicates that visual electrophysiological and pathological impairments precede overt behavioral manifestations, regardless of the relative contributions of motor deficits, sensory impairment, or anxiety to the behavioral outcomes observed at 6 months. Second, pSer129 immunoreactivity alone does not unequivocally indicate the presence of amyloid aggregates; additional markers (e.g., Thioflavin S, ubiquitin, p62) are required to confirm the aggregated nature of α‐syn inclusions. However, in our experiment, no specific staining was observed in the PBS‐injected control group under the same conditions, supporting the specificity of the signal. Third, downregulation of TH expression may reflect either actual DN loss or downregulation of TH expression in surviving neurons. In the present study, we did not distinguish between these possibilities. Future studies using pan‐neuronal markers (e.g., NeuN) or Nissl staining will be necessary to determine whether the observed reduction in TH^+^ signals correspond to irreversible neuronal degeneration or reversible phenotypic downregulation.

In the α‐syn PFF‐induced striatal injection mouse model, we observed significant visual electrophysiological abnormalities, downregulation of TH expression in retina, and activation of microglia and Müller glia prior to the onset of motor deficits. Additionally, pathological *p*‐α‐syn accumulation was detected in the retina, VC, and visual association cortices. These findings indicate that both functional and pathological alterations in the visual pathway are already present during the prodromal stage of PD, suggesting that assessments of visual pathway dysfunction and α‐syn pathology may hold potential value for early auxiliary diagnosis. Furthermore, the involvement of both retinal and central structures in visual dysfunction highlights the interplay between the eye and brain in PD pathogenesis. This model successfully recapitulates not only the motor symptoms but also the hallmark pathological features of PD, including progressive α‐synucleinopathy and DN loss, making it a valuable tool for studying chronic, sporadic PD‐related visual pathway alterations.

## Author Contributions

**Qin Zhang**: conceptualization. **Xin‐Yi Qian**: conceptualization. **Tao Peng**: conceptualization. **Xiao‐Ou Hou**: conceptualization. **Zhen‐Yuan Ma**: conceptualization. **Jiang Huang**: conceptualization. **Li‐Fang Hu**: conceptualization. **Wei‐Feng Luo**: conceptualization.

## Funding

This work was supported by grants from the Clinical Research Center of Neurological Disease fund project of the Second Affiliated Hospital of Soochow University (ND2022B06), Suzhou Science and Technology Development Plan project (SKY2022157), Project of Suzhou Health Information and Healthcare Big Data Society(MIA202401), and Discipline Construction Support Project of the Second Affiliated Hospital of Soochow University (XKTJ‐XK202412).

## Ethics Statement

All experimental procedures adhered to the ARVO Statement for the Use of Animals in Ophthalmic and Vision Research and were approved by the Institutional Animal Care and Use Committee of Soochow University.

## Conflicts of Interest

The authors declare no conflicts of interests.

## Supporting information



Fig. S1. Full‐length Western blot images.

## Data Availability

All data supporting the findings of this study are available from the corresponding author upon reasonable request.

## References

[brb371489-bib-0001] Aras, S. , G. Tanriover , M. Aslan , P. Yargicoglu , and A. Agar . 2014. “The Role of Nitric Oxide on Visual‐Evoked Potentials in MPTP‐Induced Parkinsonism in Mice.” Neurochemistry International 72: 48–57. 24795109 10.1016/j.neuint.2014.04.014

[brb371489-bib-0002] Armstrong, R. A. 2017. “Visual Dysfunction in Parkinson's Disease.” International Review of Neurobiology 134: 921–946. 28805589 10.1016/bs.irn.2017.04.007

[brb371489-bib-0003] Arrigo, A. , A. Calamuneri , D. Milardi , et al. 2017. “Visual System Involvement in Patients With Newly Diagnosed Parkinson Disease.” Radiology 285: 885–895. 28696183 10.1148/radiol.2017161732

[brb371489-bib-0004] Assogna, F. , C. Pellicano , C. Savini , et al. 2020. “Drug Choices and Advancements for Managing Depression in Parkinson`s Disease.” Current Neuropharmacology 18: 277–287. 31622207 10.2174/1570159X17666191016094857 PMC7327944

[brb371489-bib-0005] Baik, K. , Y. J. Kim , M. Park , et al. 2024. “Functional Brain Networks of Minor and Well‐Structured Major Hallucinations in Parkinson's Disease.” Movement Disorders 39: 318–327. 38140793 10.1002/mds.29681

[brb371489-bib-0006] Batum, M. , A. K. Ak , M. S. Arı , H. Mayali , E. Kurt , and D. Selçuki . 2022. “Evaluation of the Visual System With Visual Evoked Potential and Optical Coherence Tomography in Patients With Idiopathic Parkinson's Disease and With Multiple System Atrophy.” Documenta Ophthalmologica 145: 99–112. 35881261 10.1007/s10633-022-09887-7

[brb371489-bib-0007] Beach, T. G. , J. Carew , G. Serrano , et al. 2014. “Phosphorylated α‐Synuclein‐Immunoreactive Retinal Neuronal Elements in Parkinson's Disease Subjects.” Neuroscience Letters 571: 34–38. 24785101 10.1016/j.neulet.2014.04.027 PMC4591751

[brb371489-bib-0008] Bodis‐Wollner, I. , P. B. Kozlowski , S. Glazman , and S. Miri . 2014. “α‐Synuclein in the Inner Retina in Parkinson Disease.” Annals of Neurology 75: 964–966. 24816946 10.1002/ana.24182

[brb371489-bib-0011] Çevik, Ö. S. , D. D. Yıldırım , C. Uzun , et al. 2025. “Contribution of Distinctive Outcome Measures to the Assessment of Anxiety in the Open Field: A Meta‐Analysis of Factors Mediating Open‐Field Test Variability in Rodent Models of Anxiety.” Behavioural Brain Research 490: 115612. 40311939 10.1016/j.bbr.2025.115612

[brb371489-bib-0009] Cao, Q. , S. Yang , X. Wang , et al. 2024. “Transport of β‐Amyloid From Brain to Eye Causes Retinal Degeneration in Alzheimer's Disease.” Journal of Experimental Medicine 221: e20240386. 39316084 10.1084/jem.20240386 PMC11448872

[brb371489-bib-0010] Casciano, F. , E. Zauli , C. Celeghini , et al. 2024. “Retinal Alterations Predict Early Prodromal Signs of Neurodegenerative Disease.” International Journal of Molecular Sciences 25: 1689. 38338966 10.3390/ijms25031689 PMC10855697

[brb371489-bib-0012] Chakraborty, R. , T. Nonaka , M. Hasegawa , and C. Zurzolo . 2023. “Tunnelling Nanotubes Between Neuronal and Microglial Cells Allow Bi‐Directional Transfer of α‐Synuclein and Mitochondria.” Cell Death & Disease 14: 329. 37202391 10.1038/s41419-023-05835-8 PMC10195781

[brb371489-bib-0013] Choi, E. Y. , S.‐L. Ding , and S. N. Haber . 2017. “Combinatorial Inputs to the Ventral Striatum From the Temporal Cortex, Frontal Cortex, and Amygdala: Implications for Segmenting the Striatum.” eNeuro 4: ENEURO.0392–17.2017. 29279863 10.1523/ENEURO.0392-17.2017 PMC5740454

[brb371489-bib-0015] Dinter, E. , T. Saridaki , L. Diederichs , H. Reichmann , and B. H. Falkenburger . 2020. “Parkinson's Disease and Translational Research.” Translational Neurodegeneration 9: 43. 33256849 10.1186/s40035-020-00223-0 PMC7708097

[brb371489-bib-0016] Duan, Q. , Q. Zhang , K. Nie , et al. 2023. “Myo1d Promotes Alpha‐Synuclein Transfer From Brain Microvascular Endothelial Cells to Pericytes Through Tunneling Nanotubes.” iScience 26: 107458. 37575183 10.1016/j.isci.2023.107458 PMC10416064

[brb371489-bib-0017] Eastlake, K. , W. D. B. Lamb , J. Luis , P. T. Khaw , H. Jayaram , and G. A. Limb . 2021. “Prospects for the Application of Müller Glia and Their Derivatives in Retinal Regenerative Therapies.” Progress in Retinal and Eye Research 85: 100970. 33930561 10.1016/j.preteyeres.2021.100970

[brb371489-bib-0018] Ermini, F. , V. F. Low , J. J. Song , et al. 2024. “Ultrastructural Localization of *Porphyromonas gingivalis* Gingipains in the Substantia Nigra of Parkinson's Disease Brains.” npj Parkinson's Disease 10: 90. 10.1038/s41531-024-00705-2 PMC11045759 38664405

[brb371489-bib-0019] Erramuzpe, A. , A. Murueta‐Goyena , A. Jimenez‐Marin , et al. 2025. “Amygdala Neurodegeneration: A Key Driver of Visual Dysfunction in Parkinson's Disease.” Annals of Clinical and Translational Neurology 12: 768–779. 39957584 10.1002/acn3.70007 PMC12040507

[brb371489-bib-0020] Guo, L. , E. M. Normando , P. A. Shah , L. de Groef , and M. F. Cordeiro . 2018. “Oculo‐Visual Abnormalities in Parkinson's Disease: Possible Value as Biomarkers.” Movement Disorders 33: 1390–1406. 30311977 10.1002/mds.27454

[brb371489-bib-0021] Guo, M. , J. Wang , Y. Zhao , et al. 2020. “Microglial Exosomes Facilitate α‐Synuclein Transmission in Parkinson's Disease.” Brain 143: 1476–1497. 32355963 10.1093/brain/awaa090 PMC7241957

[brb371489-bib-0022] Hamedani, A. G. , D. S. Abraham , M. G. Maguire , and A. W. Willis . 2020. “Visual Impairment Is More Common in Parkinson's Disease and Is a Risk Factor for Poor Health Outcomes.” Movement Disorders 35: 1542–1549. 32662528 10.1002/mds.28182 PMC8183672

[brb371489-bib-0023] Harrison, P. J. , and S. Luciano . 2021. “Incidence of Parkinson's Disease, Dementia, Cerebrovascular Disease and Stroke in Bipolar Disorder Compared to Other Psychiatric Disorders: An Electronic Health Records Network Study of 66 Million People.” Bipolar Disorders 23: 454–462. 33075191 10.1111/bdi.13022

[brb371489-bib-0024] Hart de Ruyter, F. J. , T. H. J. Morrema , J. den Haan , et al. 2023. “α‐Synuclein Pathology in Post‐Mortem Retina and Optic Nerve Is Specific for α‐Synucleinopathies.” npj Parkinson's Disease 9: 124. 10.1038/s41531-023-00570-5 PMC10462645 37640753

[brb371489-bib-0025] Hasanov, S. , E. Demirkilinc Biler , A. Acarer , C. Akkın , Z. Colakoglu , and O. Uretmen . 2019. “Functional and Morphological Assessment of Ocular Structures and Follow‐Up of Patients With Early‐Stage Parkinson's Disease.” International Ophthalmology 39: 1255–1262. 29744762 10.1007/s10792-018-0934-y

[brb371489-bib-0026] Haug, B. A. , C. Trenkwalder , G. B. Arden , W. H. Oertel , and W. Paulus . 1994. “Visual Thresholds to Low‐Contrast Pattern Displacement, Color Contrast, and Luminance Contrast Stimuli in Parkinson's Disease.” Movement Disorders 9: 563–570. 7990852 10.1002/mds.870090510

[brb371489-bib-0027] Heermann, S. 2017. “Neuroanatomy of the Visual Pathway.” Klinische Monatsblatter fur Augenheilkunde 234: 1327–1333. 29155433 10.1055/s-0043-118101

[brb371489-bib-0028] Ho, C.‐Y. , J. C. Troncoso , D. Knox , W. Stark , and C. G. Eberhart . 2014. “Beta‐Amyloid, Phospho‐Tau and Alpha‐Synuclein Deposits Similar to Those in the Brain Are Not Identified in the Eyes of Alzheimer's and Parkinson's Disease Patients.” Brain Pathology 24: 25–32. 23714377 10.1111/bpa.12070 PMC3976129

[brb371489-bib-0029] Horan‐Portelance, L. , M. Iba , D. J. Acri , J. R. Gibbs , and M. R. Cookson . 2026. “Imaging Spatial Transcriptomics Reveals Molecular Patterns Underlying Accumulation of p‐Ser129 α‐Synuclein in a Transgenic Mouse Model.” npj Parkinson's Disease 12: 36. 10.1038/s41531-025-01246-y PMC12868666 41545426

[brb371489-bib-0030] Huang, Y.‐M. , and Q. Yin，Z . 2011. “Minor Retinal Degeneration in Parkinson's Disease.” Medical Hypotheses 76: 194–196. 20933338 10.1016/j.mehy.2010.09.016

[brb371489-bib-0031] Irwin, D. J. , J. Y. Abrams , L. B. Schonberger , et al. 2013. “Evaluation of Potential Infectivity of Alzheimer and Parkinson Disease Proteins in Recipients of Cadaver‐Derived Human Growth Hormone.” JAMA Neurology 70: 462. 23380910 10.1001/jamaneurol.2013.1933 PMC3678373

[brb371489-bib-0032] Ji, S. , L. Ye , L. Zhang , D. Xu , and J. Dai . 2022. “Retinal Neurodegeneration in a Mouse Model of Green‐Light‐Induced Myopia.” Experimental Eye Research 223: 109208. 35944726 10.1016/j.exer.2022.109208

[brb371489-bib-0033] Kaehler, K. , H. Seitter , A. M. Sandbichler , et al. 2020. “Assessment of the Retina of Plp‐α‐Syn Mice as a Model for Studying Synuclein‐Dependent Diseases.” Investigative Opthalmology & Visual Science 61: 12. 10.1167/iovs.61.6.12 PMC7415298 32503050

[brb371489-bib-0034] Kim, S. , S.‐H. Kwon , T.‐I. Kam , et al. 2019. “Transneuronal Propagation of Pathologic α‐Synuclein From the Gut to the Brain Models Parkinson's Disease.” Neuron 103: 627–641.e67. 31255487 10.1016/j.neuron.2019.05.035 PMC6706297

[brb371489-bib-0035] Koss, D. J. , D. Erskine , A. Porter , et al. 2022. “Nuclear Alpha‐Synuclein Is Present in the Human Brain and Is Modified in Dementia With Lewy Bodies.” Acta Neuropathologica Communications 10: 98. 35794636 10.1186/s40478-022-01403-x PMC9258129

[brb371489-bib-0036] Lee, I. B. , E. Lee , N.‐E. Han , et al. 2024. “Persistent Enhancement of Basolateral Amygdala‐Dorsomedial Striatum Synapses Causes Compulsive‐Like Behaviors in Mice.” Nature Communications 15: 219. 10.1038/s41467-023-44322-8 PMC10774417 38191518

[brb371489-bib-0037] Li, T. , T. Liu , J. Zhang , et al. 2023. “Neurovascular Coupling Dysfunction of Visual Network Organization in Parkinson's Disease.” Neurobiology of Disease 188: 106323. 37838006 10.1016/j.nbd.2023.106323

[brb371489-bib-0038] Liu, C. , L. Qu , Q. Li , et al. 2023. “Global Brain Analysis of Minor Hallucinations in Parkinson's Disease Using EEG and MRI Data.” Frontiers in Aging Neuroscience 15: 1189621. 38298924 10.3389/fnagi.2023.1189621 PMC10828952

[brb371489-bib-0039] Lv, Q.‐K. , K.‐X. Tao , X.‐B. Wang , et al. 2023. “Role of α‐Synuclein in Microglia: Autophagy and Phagocytosis Balance Neuroinflammation in Parkinson's Disease.” Inflammation Research 72: 443–462. 36598534 10.1007/s00011-022-01676-x

[brb371489-bib-0040] Mammadova, N. , T. Baron , J. Verchère , et al. 2021. “Retina as a Model to Study In Vivo Transmission of α‐Synuclein in the A53T Mouse Model of Parkinson's Disease.” Methods in Molecular Biology 2224: 75–85. 33606207 10.1007/978-1-0716-1008-4_5

[brb371489-bib-0041] Mammadova, N. , C. M. Summers , R. D. Kokemuller , et al. 2019. “Accelerated Accumulation of Retinal α‐Synuclein (pSer129) and Tau, Neuroinflammation, and Autophagic Dysregulation in a Seeded Mouse Model of Parkinson's Disease.” Neurobiology of Disease 121: 1–16. 30218757 10.1016/j.nbd.2018.09.013

[brb371489-bib-0042] Mansuri, S. , A. Jain , R. Singh , S. Rawat , D. Mondal , and S. Raychaudhuri . 2024. “Widespread Nuclear Lamina Injuries Defeat Proteostatic Purposes of α‐Synuclein Amyloid Inclusions.” Journal of Cell Science 137: jcs261935. 38477372 10.1242/jcs.261935

[brb371489-bib-0043] Marques, A. , N. L. Taylor , D. Roquet , et al. 2022. “Structural and Functional Correlates of Hallucinations and Illusions in Parkinson's Disease.” Journal of Parkinson's Disease 12: 397–409. 10.3233/JPD-212838 34744050

[brb371489-bib-0044] Marrocco, E. , A. Indrieri , F. Esposito , et al. 2020. “α‐Synuclein Overexpression in the Retina Leads to Vision Impairment and Degeneration of Dopaminergic Amacrine Cells.” Scientific Reports 10: 9619. 32541823 10.1038/s41598-020-66497-6 PMC7295803

[brb371489-bib-0045] Mason, D. M. , N. Nouraei , D. B. Pant , et al. 2016. “Transmission of α‐Synucleinopathy From Olfactory Structures Deep Into the Temporal Lobe.” Molecular Neurodegeneration 11: 49. 27363576 10.1186/s13024-016-0113-4 PMC4929736

[brb371489-bib-0046] Mason, D. M. , Y. Wang , T. N. Bhatia , et al. 2019. “The Center of Olfactory Bulb‐Seeded α‐Synucleinopathy Is the Limbic System and the Ensuing Pathology Is Higher in Male Than in Female Mice.” Brain Pathology 29: 741–770. 30854742 10.1111/bpa.12718 PMC8028675

[brb371489-bib-0047] Millett, M. , A. Comite , E. M. Castosa , et al. 2025. “Pathological α‐Synuclein Perturbs Nuclear Integrity.” Neurobiology of Disease 214: 107028. 40669776 10.1016/j.nbd.2025.107028 PMC12959962

[brb371489-bib-0048] Nguyen‐Ba‐Charvet, K. T. , and A. Chédotal . 2014. “Development of Retinal Layers.” Comptes Rendus Biologies 337: 153–159. 24702841 10.1016/j.crvi.2013.11.010

[brb371489-bib-0049] Nishio, Y. , K. Yokoi , K. Hirayama , et al. 2018. “Defining Visual Illusions in Parkinson's Disease: Kinetopsia and Object Misidentification Illusions.” Parkinsonism & Related Disorders 55: 111–116. 29891431 10.1016/j.parkreldis.2018.05.023

[brb371489-bib-0050] Normando, E. M. , B. M. Davis , L. de Groef , et al. 2016. “The Retina as an Early Biomarker of Neurodegeneration in a Rotenone‐Induced Model of Parkinson's Disease: Evidence for a Neuroprotective Effect of Rosiglitazone in the Eye and Brain.” Acta Neuropathologica Communications 4: 1–15. 27535749 10.1186/s40478-016-0346-z PMC4989531

[brb371489-bib-0053] Østergaard, F. G. , M. M. Himmelberg , B. Laursen , H. R. Siebner , A. R. Wade , and K. V. Christensen . 2020. “Classification of α‐Synuclein‐Induced Changes in the AAV α‐Synuclein Rat Model of Parkinson's Disease Using Electrophysiological Measurements of Visual Processing.” Scientific Reports 10: 11869. 32681050 10.1038/s41598-020-68808-3 PMC7368019

[brb371489-bib-0051] Ortuño‐Lizarán, I. , T. G. Beach , G. E. Serrano , D. G. Walker , C. H. Adler , and N. Cuenca . 2018. “Phosphorylated α‐Synuclein in the Retina Is a Biomarker of Parkinson's Disease Pathology Severity.” Movement Disorders 33: 1315–1324. 29737566 10.1002/mds.27392 PMC6146055

[brb371489-bib-0052] Ortuño‐Lizarán, I. , X. Sánchez‐Sáez , P. Lax , et al. 2020. “Dopaminergic Retinal Cell Loss and Visual Dysfunction in Parkinson Disease.” Annals of Neurology 88: 893–906. 32881029 10.1002/ana.25897 PMC10005860

[brb371489-bib-0057] Pérez‐Acuña, D. , K. H. Rhee , S. J. Shin , J. Ahn , J. Y. Lee , and S. J. Lee . 2023. “Retina‐To‐Brain Spreading of α‐Synuclein After Intravitreal Injection of Preformed Fibrils.” Acta Neuropathologica Communications 11: 83. 37210559 10.1186/s40478-023-01575-0 PMC10199563

[brb371489-bib-0054] Patterson, J. R. , N. K. Polinski , M. F. Duffy , et al. 2019. “Generation of Alpha‐Synuclein Preformed Fibrils From Monomers and Use In Vivo.” Journal of Visualized Experiments 148: 59758. 10.3791/59758. PMC10337163 31205308

[brb371489-bib-0055] Paumier, K. L. , K. C. Luk , F. P. Manfredsson , et al. 2015. “Intrastriatal Injection of Pre‐Formed Mouse α‐Synuclein Fibrils Into Rats Triggers α‐Synuclein Pathology and Bilateral Nigrostriatal Degeneration.” Neurobiology of Disease 82: 185–199. 26093169 10.1016/j.nbd.2015.06.003 PMC4640952

[brb371489-bib-0056] Pei, C.‐S. , X.‐O. Hou , Z.‐Y. Ma , et al. 2024. “α‐Synuclein Disrupts Microglial Autophagy Through STAT1‐Dependent Suppression of Ulk1 Transcription.” Journal of Neuroinflammation 21: 275. 39462396 10.1186/s12974-024-03268-4 PMC11515151

[brb371489-bib-0058] Poveda, S. , X. Arellano , O. Bernal‐Pacheco , and A. Valencia López . 2024. “Structural Changes in the Retina as a Potential Biomarker in Parkinson's Disease: An Approach From Optical Coherence Tomography.” Frontiers in Neuroimaging 3: 1340754. 38496013 10.3389/fnimg.2024.1340754 PMC10940411

[brb371489-bib-0059] Scheiblich, H. , C. Dansokho , D. Mercan , et al. 2021. “Microglia Jointly Degrade Fibrillar Alpha‐Synuclein Cargo by Distribution Through Tunneling Nanotubes.” Cell 184: 5089–5106.e21. 34555357 10.1016/j.cell.2021.09.007 PMC8527836

[brb371489-bib-0080] Sharma, A. , A. Mannan , and T. G. Singh . 2025. “Rethinking Parkinson's: The role of proteostasis networks and autophagy in disease progression.” Molecular and Cellular Neuroscience 134: 104023. 10.1016/j.mcn.2025.104023. 40490236

[brb371489-bib-0060] Shulman, J. M. , P. L. de Jager , and M. B. Feany . 2011. “Parkinson's Disease: Genetics and Pathogenesis.” Annual Review of Pathology: Mechanisms of Disease 6: 193–222. 10.1146/annurev-pathol-011110-130242 21034221

[brb371489-bib-0014] da Silva, B. R. , L. E. Santos , R. A. de Melo Reis , et al. 2019. “Müller Cells Derived From Adult Chicken and Mouse Retina Neurospheres Acquire the Dopaminergic Phenotype.” Cellular and Molecular Neurobiology 39: 99–109. 30430378 10.1007/s10571-018-0636-z PMC11469868

[brb371489-bib-0061] Sipos‐Lascu, D. , Ş. C. Vesa , N.‐C. Draghici , et al. 2024. “Decoding Visual Evoked Potential Latency: Revealing Neurological Connections in Parkinson's Disease.” Journal of Medicine and Life 17: 639. 39296437 10.25122/jml-2024-0319 PMC11407499

[brb371489-bib-0062] Song, D.‐Y. , and J.‐Y. Li . 2025. “Revisiting the Advance of Age‐Dependent α‐Synuclein Propagation and Aggregation.” Ageing and Neurodegenerative Diseases 5: 4.

[brb371489-bib-0063] Stutz, B. , F. S. L. da Conceição , L. E. Santos , et al. 2014. “Murine Dopaminergic Müller Cells Restore Motor Function in a Model of Parkinson's Disease.” Journal of Neurochemistry 128: 829–840. 24117434 10.1111/jnc.12475

[brb371489-bib-0064] Thiel, W. A. , Z. I. Blume , and D. M. J. G. Mitchell . 2022. “Compensatory Engulfment and Müller Glia Reactivity in the Absence of Microglia.” Glia 70: 1402–1425. 35451181 10.1002/glia.24182 PMC9081278

[brb371489-bib-0065] Tran, K. K. N. , V. H. Y. Wong , A. Hoang , D. I. Finkelstein , B. V. Bui , and C. T. O. Nguyen . 2023. “Retinal Alpha‐Synuclein Accumulation Correlates With Retinal Dysfunction and Structural Thinning in the A53T Mouse Model of Parkinson's Disease.” Frontiers in Neuroscience 17: 1146979. 37214398 10.3389/fnins.2023.1146979 PMC10196133

[brb371489-bib-0066] Tran, K. K. N. , V. H. Y. Wong , J. K. H. Lim , et al. 2022. “Characterization of Retinal Function and Structure in the MPTP Murine Model of Parkinson's Disease.” Scientific Reports 12: 7610. 35534594 10.1038/s41598-022-11495-z PMC9085791

[brb371489-bib-0067] Veys, L. , J. Devroye , E. Lefevere , L. Cools , M. Vandenabeele , and L. de Groef . 2021. “Characterizing the Retinal Phenotype of the Thy1‐h [A30P] α‐Syn Mouse Model of Parkinson's Disease.” Frontiers in Neuroscience 15: 726476. 34557068 10.3389/fnins.2021.726476 PMC8452874

[brb371489-bib-0068] Veys, L. , M. Vandenabeele , I. Ortuño‐Lizarán , et al. 2019. “Retinal α‐Synuclein Deposits in Parkinson's Disease Patients and Animal Models.” Acta Neuropathologica 137: 379–395. 30721408 10.1007/s00401-018-01956-z

[brb371489-bib-0069] Vidailhet, M. 2024. “Eyes as a Window to Brain Pathology in Parkinson's Disease: A Narrative Review.” Journal of Neural Transmission 131: 1155–1158. 39180544 10.1007/s00702-024-02820-z

[brb371489-bib-0070] Volpicelli‐Daley, L. A. , K. C. Luk , and V. M. Lee . 2014. “Addition of Exogenous α‐Synuclein Preformed Fibrils to Primary Neuronal Cultures to Seed Recruitment of Endogenous α‐Synuclein to Lewy Body and Lewy Neurite‐Like Aggregates.” Nature Protocols 9: 2135–2146. 25122523 10.1038/nprot.2014.143 PMC4372899

[brb371489-bib-0071] Wang, G. , P. Hou , Y. Tu , J. Zheng , P. Li , and L. Liu . 2023. “Activation of p38 MAPK Hinders the Reactivation of Visual Cortical Plasticity in Adult Amblyopic Mice.” Experimental Eye Research 236: 109651. 37748716 10.1016/j.exer.2023.109651

[brb371489-bib-0072] Wang, Q. ,Song,S.,Jiang,L., et al. 2021. “Interplay Among Norepinephrine, NOX2, and Neuroinflammation: Key Players in Parkinson's Disease and Prime Targets for Therapies.” Ageing and Neurodegenerative Diseases 1: 6.

[brb371489-bib-0079] Weil, R. S. , A. E. Schrag , J. D. Warren , S. J. Crutch , A. J. Lees , and H. R. Morris . 2016. “Visual dysfunction in Parkinson's disease.” Brain 139, no. 11: 2827–2843. 10.1093/brain/aww175. 27412389 PMC5091042

[brb371489-bib-0074] Xia, Y. , G. Zhang , C. Han , et al. 2019. “Microglia as Modulators of Exosomal Alpha‐Synuclein Transmission.” Cell Death & Disease 10: 174. 30787269 10.1038/s41419-019-1404-9 PMC6382842

[brb371489-bib-0075] Xu, T. , X. Liu , X. Lin , et al. 2024. “Abnormal α‐Synuclein Aggregates Cause Synaptic‐ and Microcircuit‐Specific Deficits in the Retinal Rod Pathway.” American Journal of Pathology 194: 796–809. 38395146 10.1016/j.ajpath.2024.01.017

[brb371489-bib-0076] Yao, N. , R. Shek‐Kwan Chang , C. Cheung , et al. 2014. “The Default Mode Network Is Disrupted in Parkinson's Disease With Visual Hallucinations.” Human Brain Mapping 35: 5658–5666. 24985056 10.1002/hbm.22577 PMC4657500

[brb371489-bib-0078] Zhou, H. , J. Su , X. Hu , et al. 2020. “Glia‐To‐Neuron Conversion by CRISPR‐CasRx Alleviates Symptoms of Neurological Disease in Mice.” Cell 181: 590–603.e16. 32272060 10.1016/j.cell.2020.03.024

