# Supplementary material for: Retinal Pathology and Synucleinopathy in the Visual Pathway of α‐Synuclein Preformed Fibril Mouse Model of Parkinson's Disease
Source: Brain Behav. 2026 May 14;16(5):e71489. doi: 10.1002/brb3.71489 (PMC13175198; doi:10.1002/brb3.71489)
Supplement: Supplementary file 1 — Fig. S1. Full‐length Western blot images. [file BRB3-16-e71489-s001.docx]

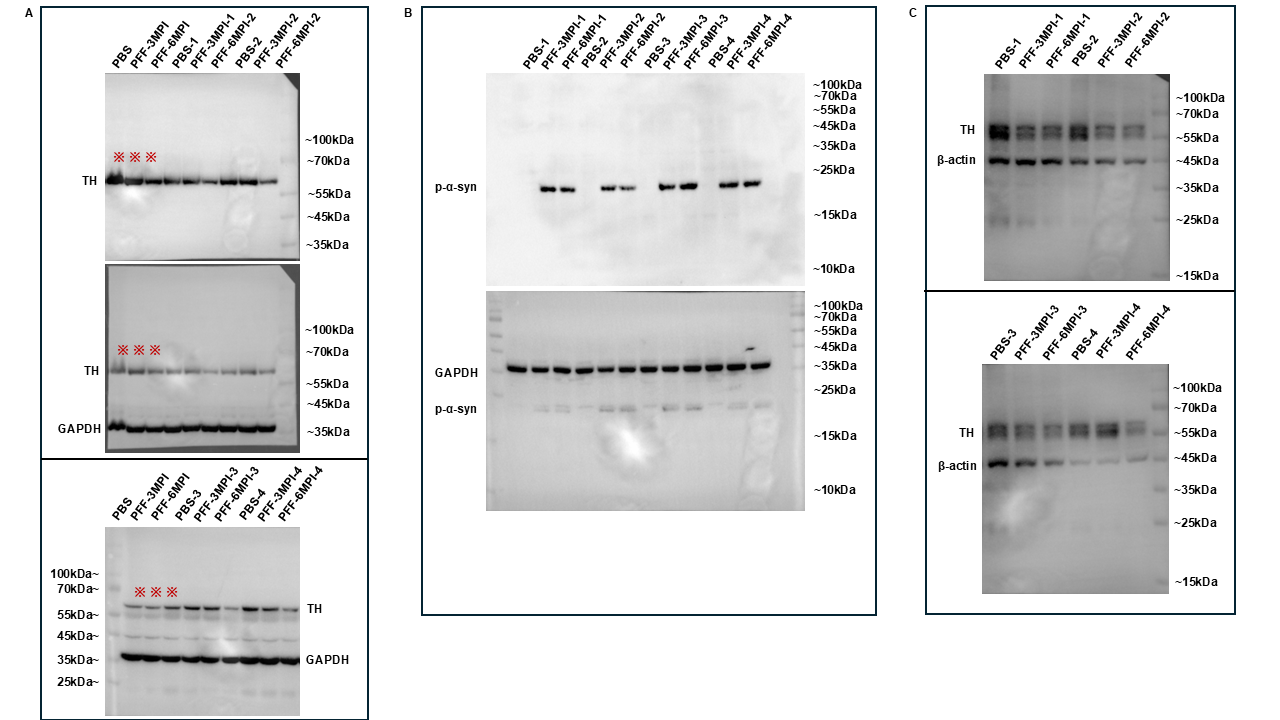


**Fig. S1.** Full-length Western blot images. (A) Representative TH blots from the substantia nigra are shown in Fig.2E. Samples excluded from the final statistical analysis due to technical issues (poor transfer quality and insufficient exposure) are indicated with a red asterisk (※). (B) Representative p-α-Syn blots from the retina are shown in Fig.4B. (C) Representative TH blots from the retina are shown in Fig.5A. PBS, n = 4; PFF-3MPI, n = 4; PFF-6MPI, n = 4.
